# Hybrid fertility and the rarity of homoploid hybrid speciation

**DOI:** 10.1093/aobpla/plaf035

**Published:** 2025-06-26

**Authors:** Harry Sanders

**Affiliations:** Department of Soil and Crop Sciences, Graduate Student, 2474 TAMU College Station, Texas A&M University, TX 77843, United States; Natural History and Conservation

**Keywords:** hybrid speciation, homoploid, pollen fertility, pollen, speciation

## Abstract

Hybrid speciation is increasingly being recognized as an important driver in diversification. Of the two forms of hybrid speciation, polyploid hybrid speciation is considered more common as it provides instant reproductive isolation either completely or partially, due to meiotic incompatibilities with the parental species. Homoploid hybrid speciation is considered rare, as it lacks the instant reproductive isolation conferred by polyploidy, though there are an increasing number of examples in the literature. Reproductive isolation in the nascent homoploid hybrid species is commonly achieved by niche divergence, often into territory with one or more forms of abiotic stress unsuitable for parental species. However, reproductive isolation may be due to factors beyond mere niche divergence. In this paper, I examine the pollen fertility of hybrids compared with their parental species. Using a dataset of over 2000 observations, I compared F1 and F2 hybrids to members of the same genus and family. F1 hybrids universally have lower mean fertility than good species in their group at both the genus and family levels, and most of those differences are significant. Data for F2 hybrids are limited and conflicting, but there may be a path to restored fertility across multiple generations such as through fertility restoration genes or selection for increased fertility. Alternatively, homoploid hybrids may rely on forms of asexual reproduction. Examples exist to support both alternatives. While pollen fertility is a useful metric, other metrics of plant fertility could also throw light on the difficulty of forming a homoploid hybrid species in plants.

## Introduction

Speciation is an important biological process that has fascinated generations of biologists. The processes by which new species form are among the most debated topics in modern biology. Opinions range from speciation being largely allopatric (Coyne and Orr 2004) to emphasizing the importance of sympatric speciation (Johannesson 2001). Some evolutionary biologists have even rejected that binary, focusing instead on the processes driving speciation (Butlin *et al.* 2008, Fitzpatrick *et al.* 2008). Botanists have commonly added that hybrid speciation by polyploidization is also common in plants (Rieseberg and Willis 2007). One review estimated at least 25% of all plants undergo some form of hybridization (Mallet 2005). The idea of hybridization among plants is not new for botanists, with experimental hybrids known as far back as the early 1700s (Raven 1977). However, not all hybridization leads to new species, so just generating new hybrids is not enough to ensure the development of a new species (Matthews *et al*. 2015). In some groups, both homoploid hybridization and polyploidy impact speciation (Winterfeld *et al.* 2016). While allopolyploid hybridization is considered much more common than homoploid hybrid speciation, there are a handful of examples of the latter in the literature (Mallet 2007).

While homoploid hybridization has long been theorized, it has been incredibly difficult to document and remains somewhat controversial. One review of the topic found just three cases of homoploid hybrid speciation met a stringent three-criteria list (Schumer *et al.* 2014). Very few instances fulfilled all three criteria (Lukhtanov *et al.* 2015), and other authors objected to how tightly drawn the criteria were (Nieto Feliner *et al.* 2017). With the advent of genomic studies, at least one case of homoploid hybrid speciation in plants has been overturned (Goulet-Scott *et al.* 2021). Other examples have been proposed, however, and their number is increasing. While somewhat controversial, homoploid hybrid speciation is still an important area of research in the plant speciation literature.

According to the theory put forward by Rieseberg (1997), homoploid hybrid speciation occurs when hybrids between two species form a new species, acquiring reproductive isolation through either spatial or ecological isolation, or chromosomal reorganization. In that review, Rieseberg listed eight examples of homoploid hybrid speciation. In a more recent paper, Rieseberg and a coauthor list two further examples of plant homoploid hybrid speciation (Long and Rieseberg 2024). Another review found 28 examples of homoploid hybrid species in plants (Kadereit 2015). Regardless of precisely how many examples of homoploid hybrid speciation exist, it appears to be a relatively rare phenomenon among plants, at least in comparison with polyploid speciation which is estimated to be roughly 15% of plant speciation events (Wood *et al.* 2009). More examples are being proposed regularly, however, including one from 2024 (Nie *et al.* 2024), so it may be possible eventually to estimate how common homoploid hybrid speciation is.

When hybrids form, it is due to the overlap of geographical ranges and phenology of two distinct species. The offspring of those hybrids must somehow become reproductively isolated from both parental species. Such isolation need not be complete, but it should be fairly strong to allow the formation of a new species. While reproductive isolation is instant in allopolyploids, it is not in homoploid hybrids (Goulet *et al*. 2017). Introgression, due to repeated backcrossing with one or both parents, can be a significant potential roadblock to homoploid hybrid speciation (López-Caamal and Tovar-Sánchez 2014). This problem is easier to overcome in plants than animals as plants often produce more offspring than animals, but it remains a difficulty.

Pollen fertility serves as a major postzygotic barrier to reproduction. In *Fragaria* hybrids, hybrid sterility, not germination or survival, provided the limits to hybridization (Nosrati *et al.* 2011). Hybrid male sterility is commonly observed in other plant genera as well (Carrió and Güemes 2014, Ferriol *et al.* 2023). Even in species with strong prezygotic isolation, postzygotic isolation mechanisms can still stymie hybrid success (Larcombe *et al.* 2016). When hybridization occurs, postzygotic isolation via reduced pollen fertility can arise very rapidly (Gustafsson *et al.* 2022). As one of the major drivers of postzygotic isolation, pollen fertility can thus play an important role in developing reproductive isolation, a key factor in speciation.

Temporary reproductive isolation may be conferred on hybrids by the low pollen fertility often associated with hybridization (Vallejo-Marin and Hiscock 2016). In fact, low pollen fertility in hybrids is sometimes considered evidence of isolation between the parents and hybrids (Rieseberg 1997). Reproductive isolation acquired in this way will only work either if there is an alternative reproductive pathway, or if pollen fertility is restored rapidly. The latter is suggested by the behaviour of synthetic hybrids between *Helianthus annuus* and *Helianthus petiolaris*, believed the ancestral species of *Helianthus anomalus*. F1 hybrids have a fertility of 0.056, but, by the F4, reach 0.918 (Ungerer *et al*. 1998). The mean pollen fertility for *H. anomalus* is 0.945 (Lai *et al*. 2005).

In a simulation study of homoploid hybrid speciation, speciation occurred quickly when the fertility of the hybrid offspring is similar to the parent, but more slowly when hybrid fertility is low (McCarthy *et al.* 1995). However, relative fertility in this simulation appears to be assumed constant, not allowing for fertility recovery. Backcrossing back to one or both parental species can relatively quickly raise fertility. In two generations of backcrossing in a *Coffea canephora* × *Coffea heterocalyx* cross, pollen viability was raised from 0.182 to 0.836, which was higher than one of the parental species (Coulibaly *et al.* 2003). The restoration process can work when the hybrids mate only with themselves as well. Three studies of good *Senecio* species (Lowe and Abbott 2004, Hodálová and Kochjarová 2006, Milton 2009) gave a mean pollen fertility of the genus of 0.915. The F1 hybrids provided in those studies had a mean pollen fertility of 0.782 and the F2 0.9.

Backcrossing, however, is not common in homoploid hybrid speciation (Marques *et al.* 2016, White *et al*. 2018) though it does occur in polyploid hybrid speciation (Ma *et al*. 2010). That means, if fertility is not restored quickly, plants must rely on other means of reproduction. Apomixis is a common way around low fertility, as most apomicts have lower fertility than sexually reproducing species (Hörandl 2024). At least one nearly sterile homoploid hybrid species uses apomixis to keep itself from extinction (Carman *et al.* 2019). However, a study in *Ranunculus* found that heteroploid, not homoploid, hybrids were associated with functional apomixis (Hojsgaard *et al.* 2014). Apomixis may thus be an option in some lineages, not others.

Apart from apomixis, some form of vegetative reproduction, or rapid fertility restoration, a hybrid may struggle to reproduce. Homoploid hybrid speciation expects the newly formed hybrids to be at least partially fertile and more fertile within the lines than with either parent (Rieseberg 1997). The fertility within the line is expected to increase over time as the incipient species stabilizes. However, the incipient species must survive to reach stabilization.

The exact way pollen fertility influences the survival of species/incipient species is unclear. Low pollination probabilities are known to influence population declines (Young *et al*. 2012), and hybrids start at very low population sizes. Poor-quality pollen is known to reduce seed production and potentially impact the offspring’s fitness (Aizen and Harder 2007). However, these are not direct measurements of pollen fertility. Instead, they measure how pollen impacts reproductive success. Pollen fertility undoubtedly contributes to poor pollen quality but may not be the only driver of it. In apomictic species, pollen fertility may not be directly relevant, but even most apomicts produce viable pollen (Falistocco *et al.* 2022, Moodley *et al.* 2024). There have been no studies of how pollen fertility impacts population survival or species extinction. However, outside of apomicts and vegetatively reproducing species, at least some level of fertility is required to keep the lineage from extinction. Lineage building and consistent phenotypes have been proposed as the definition of hybrid speciation (Hörandl 2022). An estimated 70% of plant species are considered reproductive lineages (Rieseberg *et al.* 2006). Without fertile pollen, barring pure vegetative reproduction or apomixis, a lineage will go extinct. Thus, for both outcrossing and selfing species, along with their hybrids, maintaining at least some level of pollen fertility is paramount to lineage persistence and maintaining the species.

In examples from the natural world, homoploid hybrid speciation usually does require a new niche (Mao and Wang 2011, Wang *et al*. 2022). In some cases, isolation in the new niche results in at least some genetic incompatibilities between the hybrid offspring and the parental species (Brennan *et al.* 2019). In others, specialized niche adaptations may facilitate the hybrid’s adaptation to a new habitat (Wang *et al*. 2018). However, genetic divergence between the parental species in most cases is much less than the parental species of allopolyploid hybrid species (Chapman and Burke 2007, Paun *et al.* 2009).

The objectives of this study were two-fold. The first is to determine whether pollen viability in hybrid lineages was different than that of good species. It was expected that there would be a variance between good species and their hybrids, with good species predicted to have a higher mean fertility. The second is to examine the implications of any difference in pollen fertility between hybrids and good species on the formation of new species. Because of the expected low fertility of nascent hybrids, and the need for reproductive isolation in newly forming species, homoploid hybrid species are expected to use alternative methods of reproduction, at least in the nascent phases.

## Materials and methods

For this study, a dataset was assembled using standard literature search techniques. Searches were performed with Google Scholar. Initially, the search was simply for plant hybridization studies using the keywords ‘hybrid’, ‘hybridization’, ‘pollen fertility’, and ‘pollen viability’. There were no restrictions on species, or years of study. Since ploidies were rarely available for F1 hybrids, all hybrids were used, regardless of ploidy status. Studies of cultivated crop hybrids of the same species were excluded as they did not align with the purposes of this study. Pollen fertility was recorded for both intra- and interspecific crosses. Intraspecific crosses were labelled good species crosses, and interspecific crosses were labelled F1 and F2 hybrids. If more than one method of measuring pollen fertility was included in a paper, the results were averaged. If more than one paper contained data for a given species, a weighted average was performed to obtain pollen fertility. The results were collated into a dataset in Excel.

**Figure 1.**
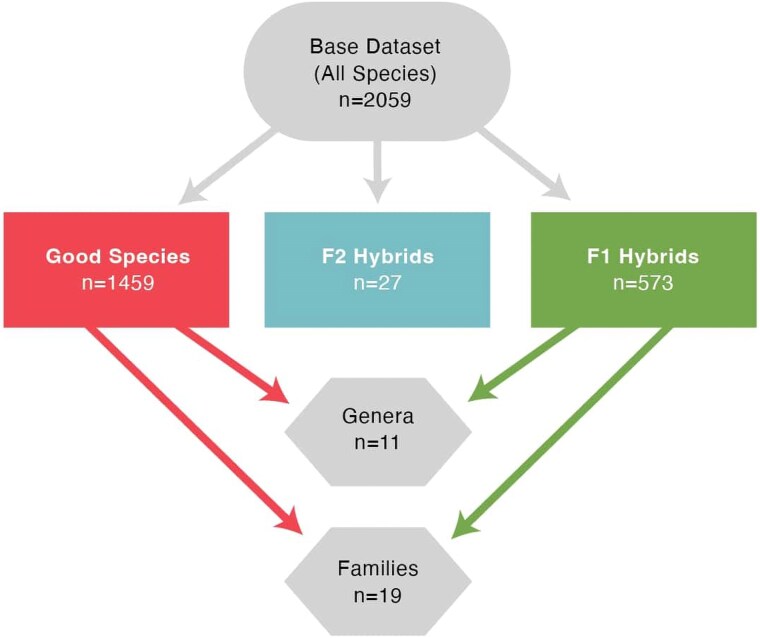
Flowchart showing how each dataset and sub-dataset were created. Note that data from the F1 and good species were combined to create the genus and family datasets.

Once a base dataset had been assembled, sub-datasets were created using the base data. Each genus listed at least once in the base dataset was queried against the literature using the same keywords as before, in addition to the genus name. All findings were used to enlarge the base dataset. Genera with a sample size of at least 10 were copied into their own datasets, resulting in eleven sub-datasets. Each genus including those broken out into separate sub-datasets was then queried against World Flora Online (WFO 2024), a database of most plant taxa, containing over 377 000 species to place it in its correct family. Using this database, a list of other genera in the family was obtained and used to query the literature as done above. Using data obtained from the literature, 19 families provided enough data for analysis. The initial dataset was also analysed, containing 1459 species and 573 hybrid combinations respectively. Figure 1 shows how each dataset was created. There were also 27 instances where hybrids were crossed to each other and their pollen fertility was examined. These were retained for analysis, though their low sample size does add uncertainty to the analysis. The final dataset consisted of over 2000 crosses (Supplementary Data S1).

**Figure 2.**
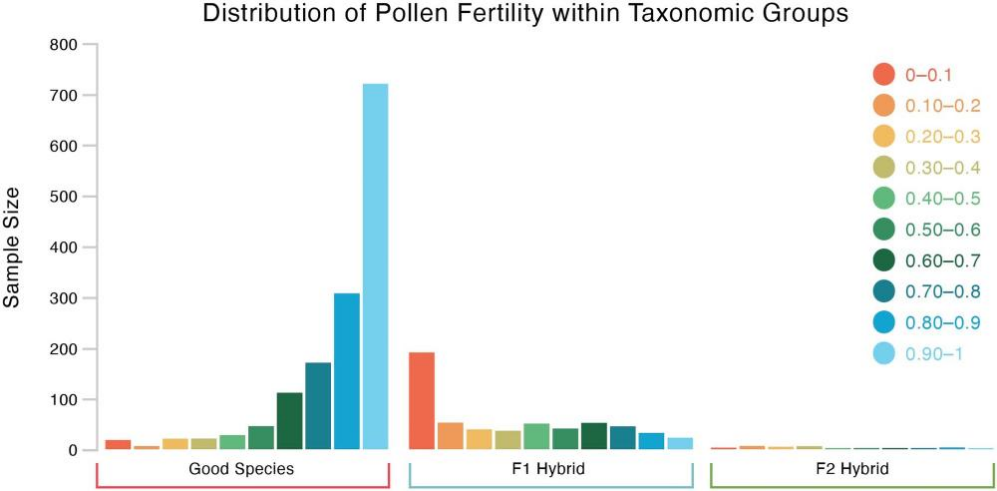
Distribution of pollen fertility in three different taxonomic groups. Note the heavy skew toward the high end in the good species and a similar skew toward the low end in F1 hybrids.

All analyses were performed using R Statistical software (v4.4.1; R Core Team 2024). A script was written using packages ‘shiny’ (Chang *et al*. 2024) and ‘tidyverse’ (Wickham *et al*. 2019) to analyse the datasets (Supplementary Data S2). A Welch *t*-test was performed comparing each group of good species to hybrids. The entire dataset was analysed first, comparing good species to hybrids. The F1 hybrids were then compared with the F2 hybrids, and the F2 hybrids were compared with the good species. Figure 2 shows the distribution of pollen fertility at the species, F1, and F2 levels. Unfortunately, no data were available beyond the F2. The dataset was then broken down at the group level, with both the genus and family levels being analysed. The families were then placed in their orders if there was more than one family per order available and analysed again.

A potentially serious limitation on these results come from Kılıç *et al* (2024), who found that most methods of pollen staining produce inflated viability percentages by staining dead pollen. Only one method, 2,3,5-triphenyltetrazolium chloride (TTC), did not stain dead pollen. A previous study in 14 *Rosa* species found that TTC was incredibly accurate. Across the 13 species where it was tested, the mean viability of unstained pollen was 0.4844. TTC measured the mean viability at 0.4868 (Wronska-Pilarek and Tomlik-Wyremblewska 2010). TTC stained the least dead pollen in a test of multiple pollen staining methods as well (Abdelgadir *et al*. 2012). However, in some cases, TTC gives slightly higher mean viabilities than other tests (Bolat and Pirlak 1999, Ilgin *et al.* 2007, Al-Najm *et al*. 2021). It may be that different plants respond differently to different tests. Regardless, because of conflicting claims, the dataset retained multiple forms of pollen viability testing.

## Results

**Figure 3.**
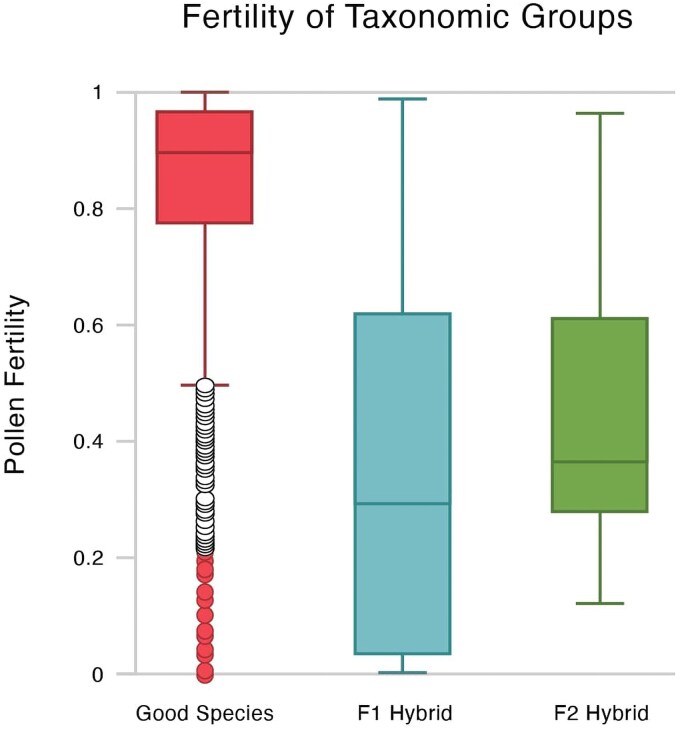
The mean of fertility among good species (*n* = 1459), F1 hybrids of good species (*n* = 573), and F2s (*n* = 27). Note that hybrids have a much broader box than good species and lack the data points outside the box. Note also that the F2 box is slightly smaller and with a lower SD than the F1. Potential outliers, including many *Hieracium* species, are labelled in red.

The results of the comparisons between good species and their F1 hybrids are remarkably consistently significant. Across the whole dataset, hybrids exhibit significantly lower pollen fertility than good species (*P* = 2.2e−16). The average pollen fertility for a good species is nearly 50% points higher than for hybrids. As shown in Fig. 3, the gap would be even larger were it not for a few outliers among good species. Many of these are *Hieracium*, an apomictic group where pollen fertility is largely irrelevant. Sixteen of the bottom 20 lowest fertility species are *Hieracium* species and the bottom 5 are all *Hieracium*.

### F1 hybrids vs F2 hybrids

Here, sample size differences may come into play as there are <30 F2 hybrid data points available, compared with 573 F1s. However, bootstrapping the data does not modify the results. F1 and F2s were not significantly different (*P* = .07). The box plot for the F2s is noticeably tighter than the F1s (Fig. 3) and the F2s have a lower standard deviation than the F1s although the lower standard deviation may be due largely to the difference in sample size.

### F2 vs good species

**Figure 4.**
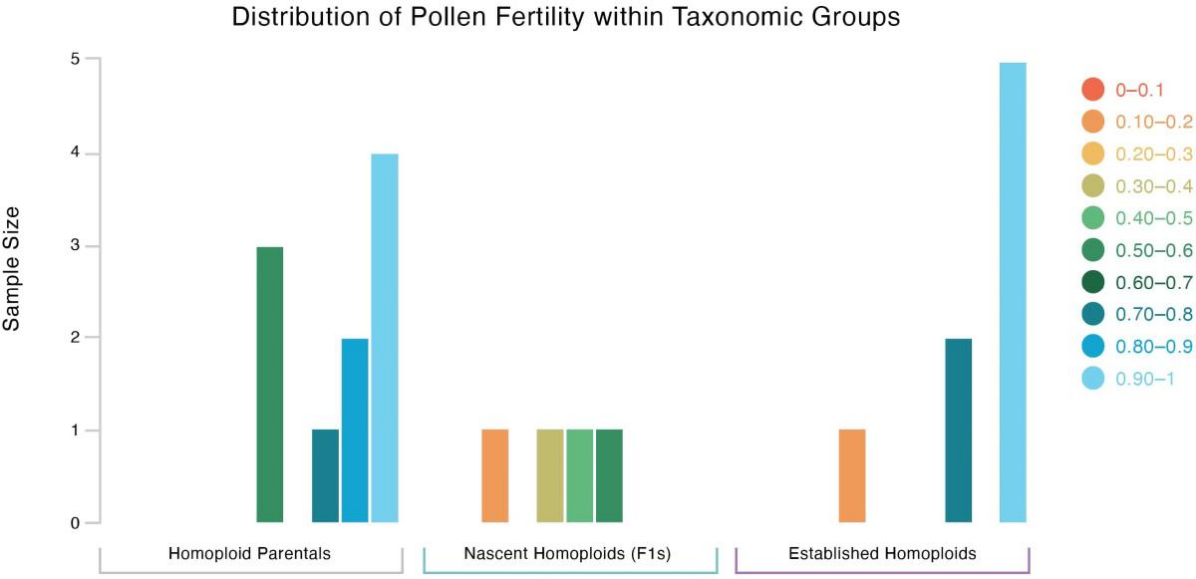
The distribution of pollen fertility in homoploid hybrid species, their parental species, and the F1 crosses leading to the homoploid species. Note the strong skew towards high pollen fertility present in both the parental and homoploid species, but absent in the F1s.

The F2 was then compared with the true species. The F2s had lower average fertility by just under 40 points and were significantly different from the true species (*P* = 1.034e−7). The mean was, however, greater than that of F1 hybrids. The mean of each taxonomic level is shown in Fig. 4.

### Genera

For most genera, there were not enough data to make comparisons at the F2 level. Thus, genus-level data only reflect F1 crosses except for *Dactylis* and *Senecio.* The results for each genus are given in Table 1. In every instance, hybrid fertility was lower than that of good species. The difference in means across all examined genera was over 40 points. Two genera with more than 10 data points had to be excluded for lacking either sufficient species or sufficient hybrid data.

**Table 1.** List of genera for which pollen fertility data were available and mean pollen fertility for the hybrids and good species in the genus.

Genus	Number of hybrids	Number of good species	Mean hybrid fertility	Mean species fertility	*P*-value
*Gillia*	61	15	0.15	0.74	4.038e−9^*^
*Senecio*	2	15	0.78	0.92	.6063
*Solanum*	7	17	0.41	0.86	.01203^*^
*Fuschia*	11	13	0.18	0.72	7.67e−6^*^
*Elymus*	13	5	0.19	0.72	.04253^*^
*Helianthus*	20	11	0.33	0.96	1.69e−9^*^
*Passiflora*	2	11	0.51	0.83	.2647
*Medicago*	3	11	0.77	0.86	.05971
*Tolpis*	13	8	0.49	0.9	8.481e−5^*^
*Cucumis*	11	6	0.25	0.7	2.121e−5^*^
*Dactylis*	18	8	0.49	0.86	.01203^*^
Mean	14.64	10.91	0.393	0.82	N/A

Note that the *Dactylis* and *Senecio* species—F2 and F1–F2 results are not given in this table.

^*^Significance.

In most instances, except *Senecio*, *Passiflora*, and *Medicago*, good species had a significantly greater pollen fertility than hybrids of the same genus. These three genera had the lowest number of hybrids of any genus tested (two, two, and three, respectively). The small sample size may explain why the results were not significant. Intriguingly, in *Dactylis*, the F2 hybrids were significantly less fertile ‘than the F1 hybrids’, which were significantly less fertile than good species. However, this difference was reversed in *Senecio*, with the F2 having higher fertility than the F1 (Supplementary Table S1). However, because of *Senecio*’s low sample size, it is possible that the fertility numbers in this genus do not represent reality. See Supplementary Figs S1–S11 for the distribution of fertility in each genus.

### Families

Nineteen families had enough data for analysis. The results are given in Table 2. In most cases, the results were significant. In two cases, Rosaceae and Caryophyllaceae, the results were not significant. In Rosaceae, this may be due to the sample size of hybrids (3), compared with good species (50). A larger sample size might provide a clearer picture of pollen fertility in this family. However, Caryophyllaceae does not have such a large disparity, although there are more species than hybrids in the data. See Supplementary Figs S12–S29 for the fertility distributions in each family.

**Table 2.** List of families for which pollen fertility data were available and mean pollen fertility for the hybrids and good species in the genus.

Family group	Number of hybrids	Number of good species	Mean hybrid fertility	Mean species fertility	*P*-value
*Liliaceae*	8	22	0.22	0.79	.001145*
*Polemoniaceae*	58	22	0.15	0.75	3.367e−15*
*Solanaceae*	16	46	0.31	0.86	1.855e−6*
*Lamiaceae*	11	81	0.29	0.88	.0003317*
*Fabaceae*	28	125	0.41	0.86	2.949e−9*
*Onagraceae*	11	14	0.24	0.77	5.088e−5*
*Asteraceae*	178	308	0.36	0.80	2.2e−16*
*Poaceae*	84	225	0.28	0.85	2.2e−16*
*Saxifragaceae*	5	8	0.48	0.79	.03622*
*Brassicaceae*	31	38	0.38	0.84	1.097e−7*
*Cayophyllaceae*	8	24	0.71	0.93	.1011
*Iridaceae*	2	20	0.05	0.87	.0009016*
*Malvaceae*	7	25	0.57	0.86	.02396*
*Hydrangeaceae*	5	16	0.34	0.76	.001663*
*Curcurbitaceae*	21	14	0.35	0.83	6.89e−7*
*Ranunculaceae*	66	28	0.62	0.82	2.607e−7*
*Rosaceae*	3	50	0.5	0.76	.184
*Rubiaceae*	2	34	0.14	0.84	.007659*
*Asparagaceae*	31	30	0.51	0.86	2.72e−9*
Mean	29.7	57.05	0.369	0.8015	N/A

^*^Significance.

An additional set of analyses were performed using putative homoploid hybrid species and putative parental species. While sample sizes were small (10 parentals and 8 homoploid hybrids), there was no difference between their mean pollen fertilities. Parentals had a mean pollen fertility of 0.818 and homoploid hybrids a mean of 0.814. The homoploid hybrid species were then compared with the F1 hybrids from the entire dataset. There was an over 45% difference in mean, and this difference was significant (*P* = .0003115). There was, however, no difference between good species and homoploid hybrid species (*P* = .7797).

Homoploid hybrid species were then compared with F1 hybrids between species known to be parentals of a homoploid hybrid species. Sample size again was low (four F1 hybrids and eight homoploid hybrid species), but the F1s had a mean of 0.27, over 50% lower than the homoploid hybrid species. The difference was significant (*P* = .005474). Distribution of pollen fertility in homoploid hybrid species, their parental species, and the F1 crosses leading the homoploid species are shown in Fig. 4.

## Discussion

In every case in this analysis, pollen fertility was lower in hybrids than in good species. This is the expected result of the biological species concept (Mayr 1988), where outbreeding is generally considered deleterious and something species should strive to avoid. Outbreeding with different species may break up favourable gene combinations accumulated under selection and result in less-fit offspring, i.e. hybrids (Mayr 1996). If this is true, then one way this lower fitness might manifest would be lower gamete fertility, as shown in this paper.

In practice, if one assumes Mayr’s view of a species to be the correct one (there are many other competitors, more than can be discussed here), then this should limit the formation of hybrid species. This difficulty may be compounded by the results from the F1–F2 comparisons in *Dactylis*, which revealed a further drop in pollen fertility in the F2 compared with the F1. The F1 pollen fertility mean was 40 points lower than the parental species mean, and the F2 was a further 15 points below the F1. Unfortunately, data for any further generations are lacking, but assuming the decline does not reverse, hybrid species would struggle to start lineages.

The majority of homoploid hybrid species examined in this study have high pollen fertility (>0.9), even *Citrus ryukyuensis*, an apomictic homoploid hybrid species (Yamamoto 2023). Yet, the mean fertility among F1 hybrids was 0.393 at the genus level and lower for families. That data would hint that these examples are either outliers or have restored pollen fertility during the speciation process. In *Helianthus*, pollen fertility restoration systems are known (Vrânceanu and Stoenescu 1978, Horn *et al.* 2003), making it possible for selection to favour the more fertile members of the population, restoring fertility over time.

Fertility restoration over time is borne out by the admittedly small analysis of homoploid hybrid species and the crosses that formed them. Fertility appears to increase over time in the hybrid species, with F1s of homoploid hybrids having significantly lower pollen fertility than their descendant species. This confirms the prediction of Rieseberg (1997) that selection should increase fertility in nascent hybrid species. The exact cause of this recovery is unclear. Chromosomal rearrangements reduce fertility in the F1 as has been shown in *Carex* and *Mimulus* (Stathos and Fishman 2014, Escudero *et al.* 2016). In butterflies, these chromosomal rearrangements resolve themselves over time, with the F1s forming the most abnormal pairs, decreasing to the F4 (Lukhtanov *et al.* 2020). Three homoploid hybrid sunflowers have significant karyotype differences compared with their parents but have comparable fertility to their parents, indicating that chromosomal rearrangements occurred in the past, but that fertility was restored (Lai *et al.* 2005). This suggests that chromosomal rearrangements reduce fertility, but that, as meiosis returns to normal, fertility follows.

It is possible, however, that chromosomal rearrangements may not reduce fertility. In the sunflower example cited above, all but one QTL associated with pollen viability is associated with a chromosomal rearrangement (Lai *et al.* 2005). In a *Mimulus* hybrid, QTLs associated with male fertility were in a region where recombination was suppressed after a rearrangement (Fishman *et al.* 2013). It is possible therefore that rearrangements reduce fertility by default. The idea that at least some chromosomal rearrangements reduce fertility is not a new one (King 1987), but rapid fertility restoration as a result of corrected meiotic pairing in the subsequent generations is an area where further research is needed.

However, rearrangements alone may be insufficient to reduce fertility. If, for example, rearrangements do not involve pollen fertility genes, then it is possible that fertility could be unaffected. If fertility is lost due to mechanisms other than chromosomal rearrangements or cannot be restored by corrected meiotic pairings, the other mechanisms, like apomixis or vegetative reproduction, are required.

There is some evidence that hybridization can trigger apomixis. In the *Rubus* subgenus *Rubus*, apomictic species cross with sexual species, creating apomictic microspecies (Šarhanová *et al*. 2017). A similar process occurs in *Arabis* where sexual and apomictic species combine to produce an apomictic hybrid species (Koch *et al.* 2003). Similar results have been reported in *Ranunculus* (Paun *et al*. 2006), *Citrus* (Wu *et al*. 2021), and *Boechera* (Beck *et al.* 2011). Nascent homoploid hybrid species may be able to survive low pollen fertility if apomixis is available. In *Boechera* hybrids, F2 plants had pollen fertility as low as 0.0187, though the average was much greater (Schranz *et al.* 2006). Apomixis provides a potential escape from low fertility and a path for homoploid hybrid speciation.

Hybridization has been suggested as a potential cause for the development of vegetative reproduction (Neiman *et al*. 2014). One study found that hybridization was associated with the development of asexual reproduction, but only sometimes (Mitchell *et al.* 2019). Hybrids of two *Pitcairnia* species reproduced primarily by vegetative reproduction (Wendt *et al.* 2001). In a list of invasive plant hybrid taxa, roughly half reproduced clonally (Ellstrand and Schierenbeck 2006). In an extensive experiment with *Salix* species, hybrids were more vigorous in vegetative reproduction than all parental species, but in most cases also outperformed the parents in sexual traits as well (Salick and Pfeffer 1999). Clonal reproduction is also noted as common, but not exclusive, in two hybrid populations of *Iris* (Burke *et al.* 2000). Vegetative reproduction may therefore serve as an alternative pathway to lineage building, but only in some instances.

Both apomixis and vegetative reproduction may provide an escape from collapsing pollen fertility during hybridization events. However, pollen fertility may not collapse in all cases. In *Senecio*, though the results are not significant (likely due to low sample size), there is a 2% difference between good species and the F2. Interestingly, *Senecio squalidus* is one of the commonly cited examples of homoploid hybrid speciation (Nevado *et al.* 2020) and it is an outcrossing sexually reproducing species (Hiscock 2000), with 94.5% pollen fertility (Lowe and Abbott 2004). Its parental species, *Senecio aethnensis* and *Senecio chrysanthemifolius*, both have relatively high proportions of poor pollen, 40%, and 35% respectively, and the hybrid between them has 39% (Brennan *et al.* 2016). These numbers seem to imply low fertility relative to *S. squalidus*. However, poor pollen does not necessarily equal infertile pollen, so true fertility may be higher than indicated here.

Interestingly, *S. squalidus* is believed to have been introduced to the UK as a hybrid in 1620 (possibly beginning cultivation as later as 1690) and was described by Linnaeus in 1753 (Abbott *et al*. 2009). The timeline of merely 133 years accords well with a simulation study that showed that the closer the fertility of the newly formed hybrid was to the parent, the faster speciation occurred (McCarthy *et al.* 1995). The F1 hybrid of *S. squalidus* parental species only varies slightly from them in fertility (Brennan *et al.* 2016). *Senecio squalidus* is a perennial but a short-lived one (Brennan *et al.* 2002). Assuming a 1-year generation time, 133 generations occurred between arrival in the UK, and description by Linnaeus as a good species. That is ample time for the diversification of a new species under the aforementioned simulation model.

The difficulty for a fertile hybrid is the likelihood of backcrossing into one of the paternal populations. In the case of *S. squalidus*, hybrid specimens were taken from their native habitats to botanical gardens, from which they escaped into the wild (James and Abbott 2005). This allowed them to bypass the difficulty of backcrossing into their parental populations and establish themselves as good species, although they have hybridized extensively with native *Senecio* species to form three additional new taxa (Abbott *et al.* 2009).

Even when fertility returns to normal, there are postzygotic isolating mechanisms that may hamper hybrid speciation. In an example of closely related flycatchers, hybridization produced <3% of descendants as mating within a good species, massively reducing fitness (Wiley *et al.* 2009). Close genetic relationship appears to have little effect on postzygotic isolation in at least some plants (Nosrati *et al.* 2011). Postzygotic isolation does not prevent hybridization or backcrossing from occurring, however (Zhang *et al*. 2016). This leaves the door open for the hybrids to potentially backcross into the parental population, particularly if postzygotic isolation is not strong. In such a scenario, isolation by niche divergence may be necessary.

Homoploid hybrid speciation is complex, and pollen fertility may play a variable role, from ancillary to important. Niche divergence and the fitness of a hybrid for a new niche may play an outsized role, as it is commonly associated with homoploid hybrid speciation (Wang *et al*. 2022). Often, fitness in a hybrid involves its fertility (White *et al.* 2018), but in some cases, habitat specific adaptations are also in play (Gross and Rieseberg 2005). Often these novel adaptations are generated as a result of hybridization (Rieseberg and Carney 1998). Often, these adaptations are associated with an altitudinal gradient (Abbott and Brennan 2014). An example of this is the pine *Pinus densata*, which adapted to a different ecological niche than its parental species, leading to its proliferation (Mao and Wang 2011).

Another form of niche divergence is stress tolerance, as seen in *Helianthus paradoxus* and *Helianthus deserticola*, both homoploid hybrid species, with high pollen fertility, at 95.6% and 90.3%, respectively (Lai *et al.* 2005). *Helianthus paradoxus* exhibits niche diversification (Welch and Rieseberg 2002), potentially driven by salt tolerance genes in *H. paradoxus*, opening up its salt marsh habitat that was unavailable to its progenitors (Lexer *et al*. 2004). This was also true for *H. deserticola*, which inhabits deserts, whereas its two progenitors prefer different habitats (Gross *et al.* 2004). Like other *Helianthus* species, *H. paradoxus* and *H. deserticola* are considered unlikely to either be or become apomictic (Noyes 2007). Vegetative reproduction is known in *Helianthus* (Mandel 2010) but has not been recorded in either *H. paradoxus* or *H. deserticola*, meaning that habitat divergence was likely required for speciation.

A similar situation occurs in *Iris nelsonii*, a homoploid hybrid species, that can spread clonally (Burke *et al.* 2000), and also retains relatively high pollen fertility, circa 85% of its parental species (Taylor *et al.* 2013). It has been suggested that *I. nelsonii* is likewise divergent in habitat from its parental species (Taylor *et al.* 2011). Interestingly, all three species *H. paradoxus*, *H. deserticola*, and *I. nelsonii* are more tolerant of abiotic stress than their progenitors, though to different stressors and different degrees (Lexer *et al.* 2004, Brouillette *et al.* 2006, Taylor *et al.* 2011).

While habitat divergence is commonly associated with homoploid hybrid speciation, this is not always a requirement. In *Ostryopsis intermedia*, reproductive isolation is achieved through two premating and one postmating reproductive barrier, only one of which is a stress tolerance component (Wang *et al*. 2021). Pollinators may also introduce a reproductive barrier between hybrids and parental species if the two are primarily favoured by different pollinators (Ma *et al.* 2016). A model of homoploid hybrid speciation found that reproductive isolation can arise simply by selection against hybrid genetic incompatibilities (Schumer *et al.* 2015). This model would make it possible for nascent hybrid species to form, even when sharing the same niche as their parental species.

## Conclusion

Hybrids have lower fertility than good species on average. This accords with the original hypothesis of this paper and meets theoretical expectations. Yet that has not precluded at least some, if not much homoploid hybrid speciation from occurring in plants. Unexpectedly, fertility restoration, rather than alternative modes of reproduction, appears to be the common path followed by most homoploid hybrid species. The aforementioned examples of homoploid hybrid species are mostly outliers compared with F1 and F2 hybrids, in that they have high fertility. However, they have had many generations to restore fertility, so this is not entirely unexpected, especially given the above cited examples of rapid fertility restoration. The examples of homoploid hybrid speciation in plants may represent survivorship bias. Nascent hybrid species with low fertility may also go extinct, leaving only those with high fertility for study. However, there are multiple paths for a low-fertility nascent hybrid to achieve species status. These paths are much less common than high fertility among extant homoploid hybrid species among plants.

Further research is needed to determine whether apomixis and vegetative reproduction promote homoploid hybrid speciation. Theoretically, they provide an escape from the low pollen fertility that applies to most hybrids, but practically, most homoploid hybrid species do not seem to rely on them, either because of a preference for sexual reproduction or because pollen fertility remains high or is restored. However, it is unknown what the F1 pollen fertility of the homoploid hybrid species was. The examples available are limited and tentative. F1s appear to have low fertility, but with such a small sample size, it is important to be very tentative. Due to fertility restoration, it is possible the F1s began with low fertility and over time restored fertility and that is supported by the data in this paper. The mechanisms of such rapid fertility restoration are unclear, and an area where much more research is needed. Further research is required to determine how much fertility restoration can impact homoploid hybrid speciation.

Another area of future research is the impact of abiotic stress on homoploid hybrid speciation. Reproductive isolation is a requirement for speciation in general, but how that isolation is achieved varies. Abiotic stress tolerance is frequently associated with the development of reproductive isolation. Whether abiotic stress is a common feature or simply reflects survivorship bias in homoploid hybrid species is an area for further investigation. While low pollen fertility is a potential obstacle to homoploid hybrid speciation in plants, it is not an insurmountable one, with numerous homoploid hybrids having developed ways around it. As more examples of homoploid hybrid speciation are discovered, it may become easier to predict the pathways by which such speciation occurs.

## Supplementary Material

plaf035_Supplementary_Data

## Data Availability

All data and code used in this analysis are available as supporting information for this paper.
