# Supplementary material for: Hybrid fertility and the rarity of homoploid hybrid speciation
Source: AoB Plants. 2025 Jun 26;17(4):plaf035. doi: 10.1093/aobpla/plaf035 (PMC12269824; doi:10.1093/aobpla/plaf035)
Supplement: plaf035_Supplementary_Data [file plaf035_supplementary_data.zip › Supplemental Information 1.pdf]

```r
library(shiny)
library(tidyverse)

ui <- fluidPage(
  titlePanel("Hybrids and True Species Analysis"),
  sidebarLayout(
    sidebarPanel(
      fileInput("file_hybrids", "Upload Hybrids CSV", accept = c(".csv")),
      fileInput("file_f2", "Upload True Species CSV", accept = c(".csv")),
      selectInput("test_type", "Choose Statistical Test",
                  choices = c("t-test", "ANOVA")),
      actionButton("analyze", "Run Analysis"),
      downloadButton("download_summary", "Download Summary Data"),
      downloadButton("download_stats", "Download Statistical Results"),
      downloadButton("download_plot", "Download Boxplot")
    ),
    mainPanel(
      tableOutput("summary"),
      plotOutput("boxplot"),
      verbatimTextOutput("stats_output")
    )
  )
)

server <- function(input, output) {
  library(shiny)
  library(tidyverse)

  # Reactive data for Hybrids
  hybrids_data <- reactive({
    req(input$file_hybrids)
    df <- read.csv(input$file_hybrids$datapath)
    df$Group <- "Hybrid" # Add group identifier
    df
  })

  # Reactive data for True Species
  f2_data <- reactive({
    req(input$file_f2)
    df <- read.csv(input$file_f2$datapath)
    df$Group <- "True Species" # Add group identifier
    df
  })
```

```r
# Combine both datasets
combined_data <- reactive({
  req(hybrids_data(), f2_data())
  bind_rows(hybrids_data(), f2_data())
})

# Summary statistics
summary_data <- reactive({
  req(combined_data())
  combined_data() %>%
    group_by(Group) %>%
    summarize(
      Mean = mean(Pollen_fertility, na.rm = TRUE),
      SD = sd(Pollen_fertility, na.rm = TRUE),
      n = n(),
      .groups = "drop"
    )
})

# Render summary table
output$summary <- renderTable({
  summary_data()
})

# Boxplot rendering
plot_boxplot <- reactive({
  req(combined_data())
  ggplot(combined_data(), aes(x = Group, y = Pollen_fertility, fill = Group)) +
    geom_boxplot() +
    labs(title = "Pollen Fertility: Hybrids vs True Species",
         x = "Group", y = "Pollen Fertility") +
    theme_minimal() +
    ylim(0, 1) # Restrict y-axis to 0-1 range
})

output$boxplot <- renderPlot({
  plot_boxplot()
})

# Statistical test logic
stats_results <- reactive({
  req(combined_data(), input$analyze)
  df <- combined_data()
  if (input$test_type == "t-test") {
```

```r
      capture.output(t.test(Pollen_fertility ~ Group, data = df))
    } else if (input$test_type == "ANOVA") {
      capture.output(summary(aov(Pollen_fertility ~ Group, data = df)))
    }
  })

  # Render statistical test results
  output$stats_output <- renderPrint({
    cat(stats_results(), sep = "\n")
  })

  # Download handlers
  output$download_summary <- downloadHandler(
    filename = function() {
      paste("summary_data", Sys.Date(), ".csv", sep = "")
    },
    content = function(file) {
      write.csv(summary_data(), file, row.names = FALSE)
    }
  )

  output$download_stats <- downloadHandler(
    filename = function() {
      paste("stats_results", Sys.Date(), ".txt", sep = "")
    },
    content = function(file) {
      writeLines(stats_results(), file)
    }
  )

  output$download_plot <- downloadHandler(
    filename = function() {
      paste("boxplot", Sys.Date(), ".png", sep = "")
    },
    content = function(file) {
      ggsave(file, plot = plot_boxplot(), width = 8, height = 6)
    }
  )
}

shinyApp(ui = ui, server = server)
```
