# Supplementary material for: Hybrid fertility and the rarity of homoploid hybrid speciation
Source: AoB Plants. 2025 Jun 26;17(4):plaf035. doi: 10.1093/aobpla/plaf035 (PMC12269824; doi:10.1093/aobpla/plaf035)
Supplement: plaf035_Supplementary_Data [file plaf035_supplementary_data.zip › Supplemental Information 2.pdf]

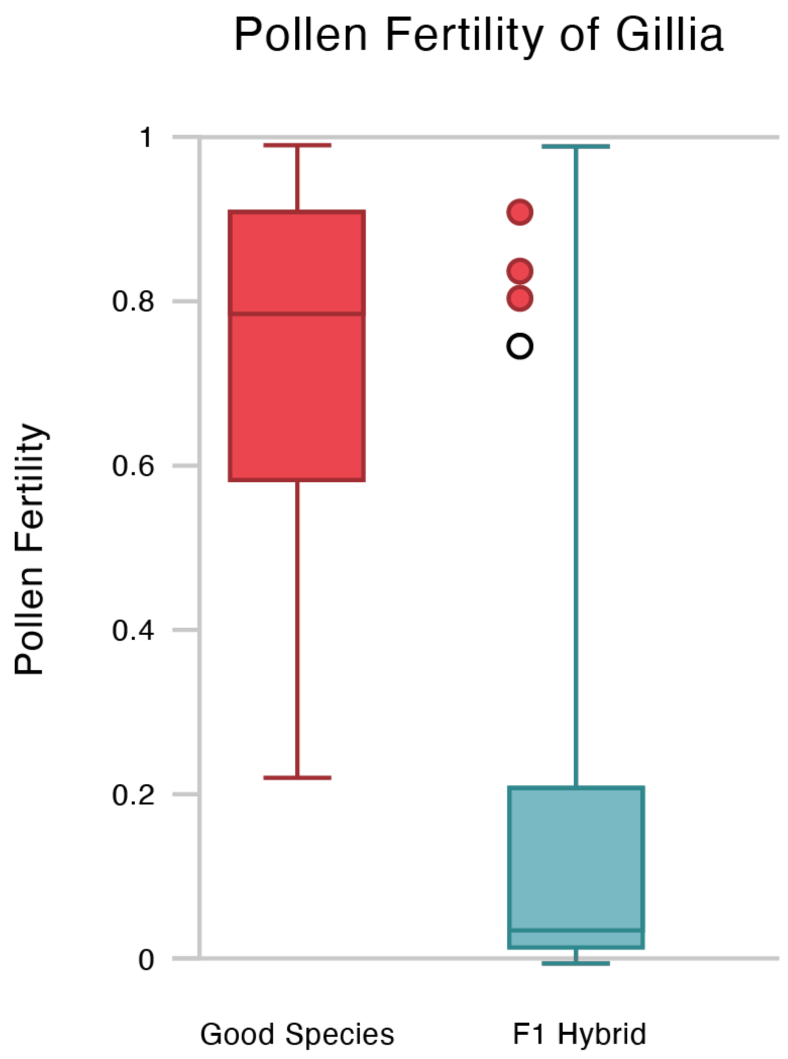

Supplementary Figure 1: *Gillia* F1 hybrids (n=58) vs good species (n=15). Note that a few *Gillia* hybrids have fertility as high, or higher than true species. These are coded as outliers, but they may represent nascent speciation.

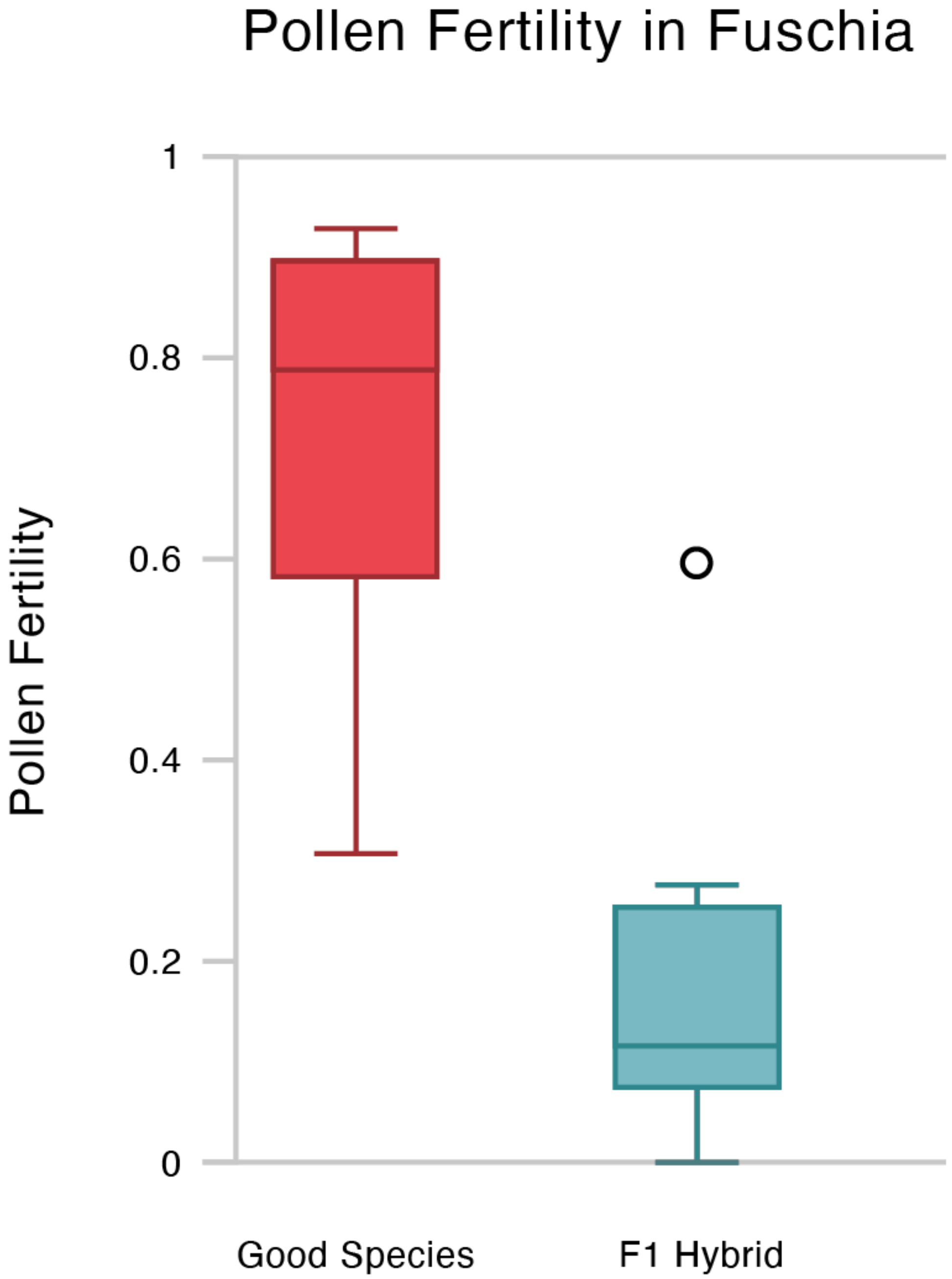

Supplementary Figure 2: *Fuschia* F1 hybrids (n=9) vs good species (n=11).

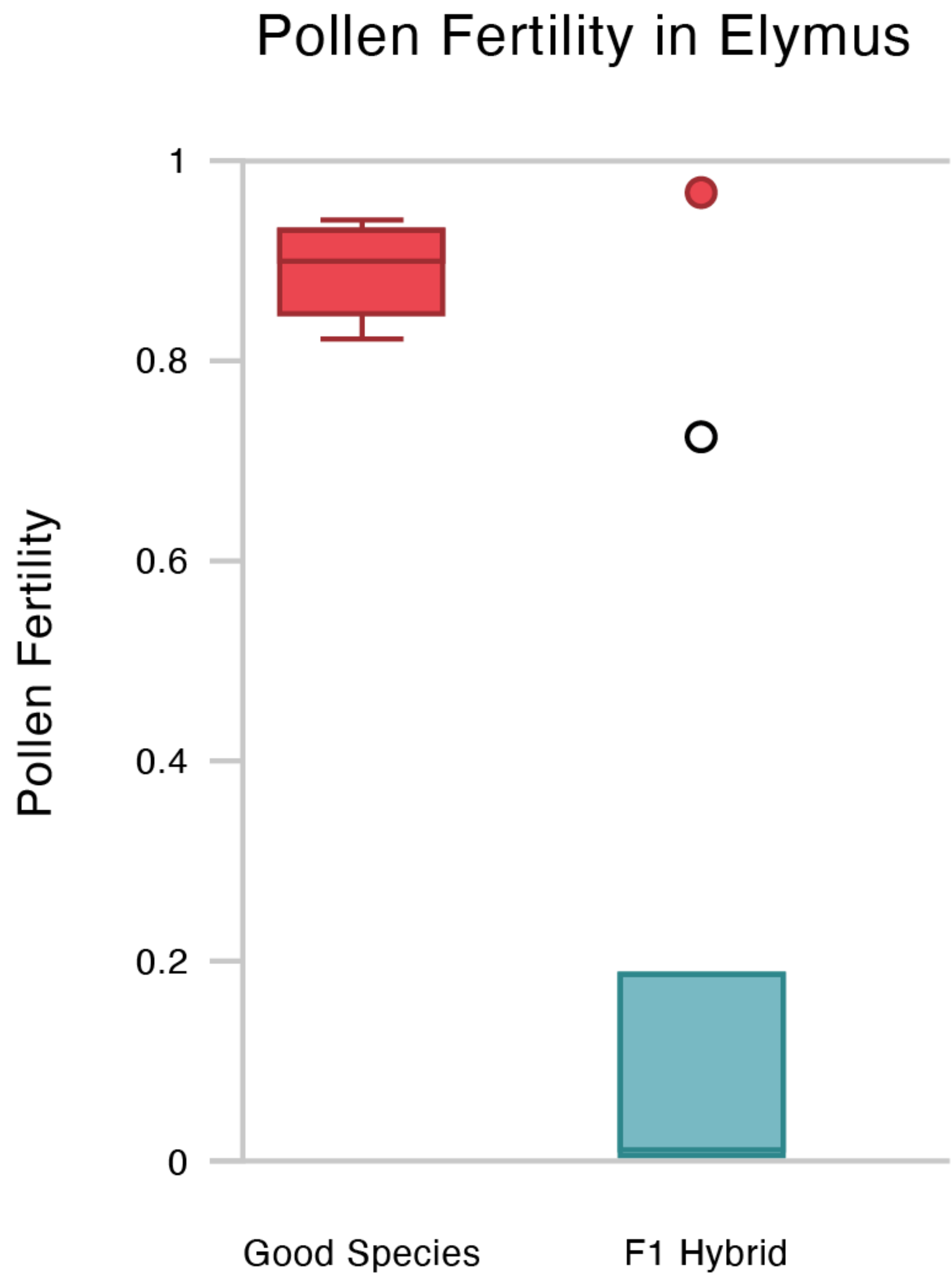

Supplementary Figure 3: *Elymus* F1 hybrids (n=9) vs good species (n=4). Note that several of the hybrids are intergeneric, which may account for the low fertility in most F1s.

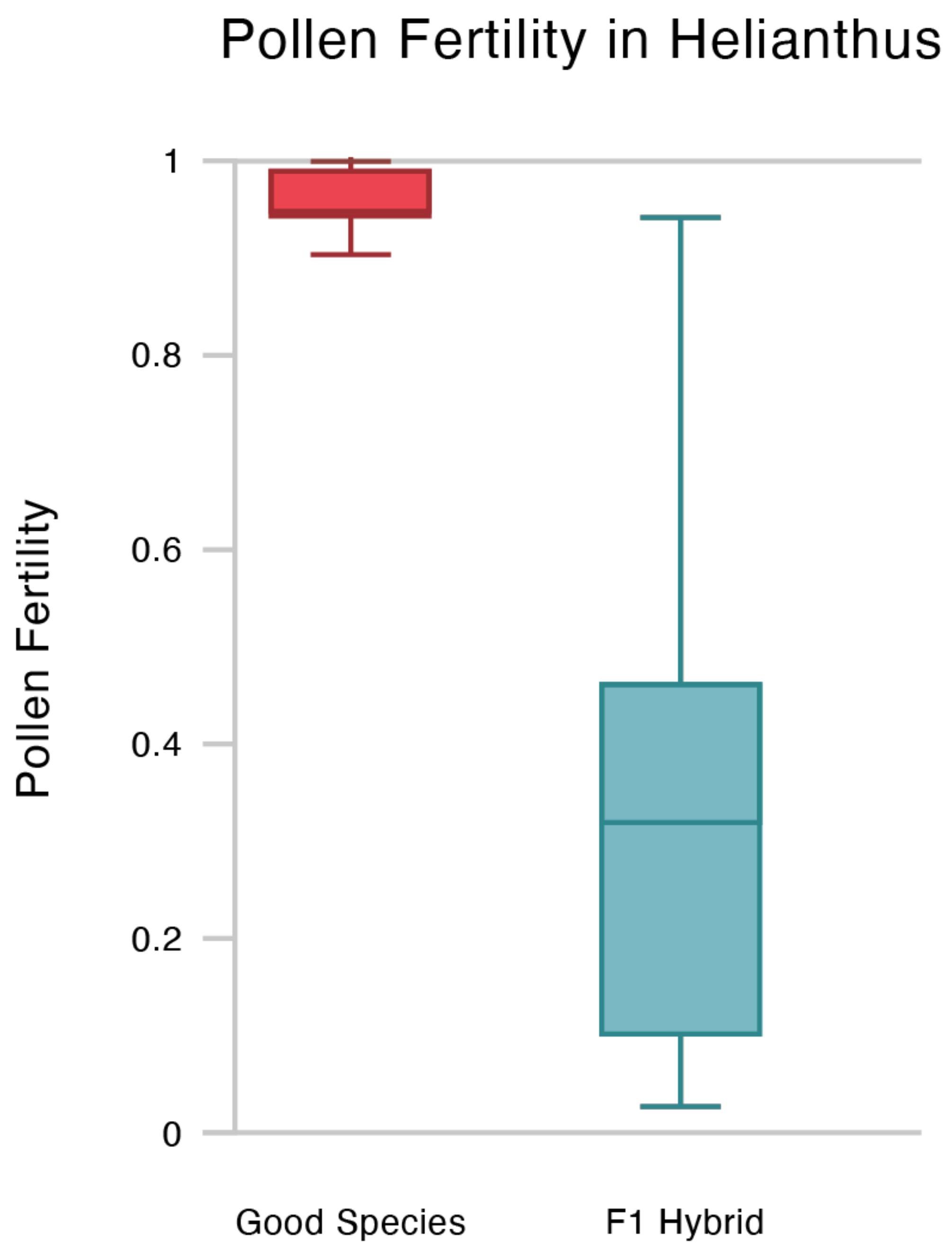

Pollen Fertility in Helianthus
Pollen Fertility
1
0.8
0.6
0.4
0.2
0
Good Species
F1 Hybrid

Supplementary Figure 4: *Helianthus* F1 hybrids (n=20) vs good species (n=11). Examples of extant species formed by homoploid hybridization are found in the good species side of the plot.

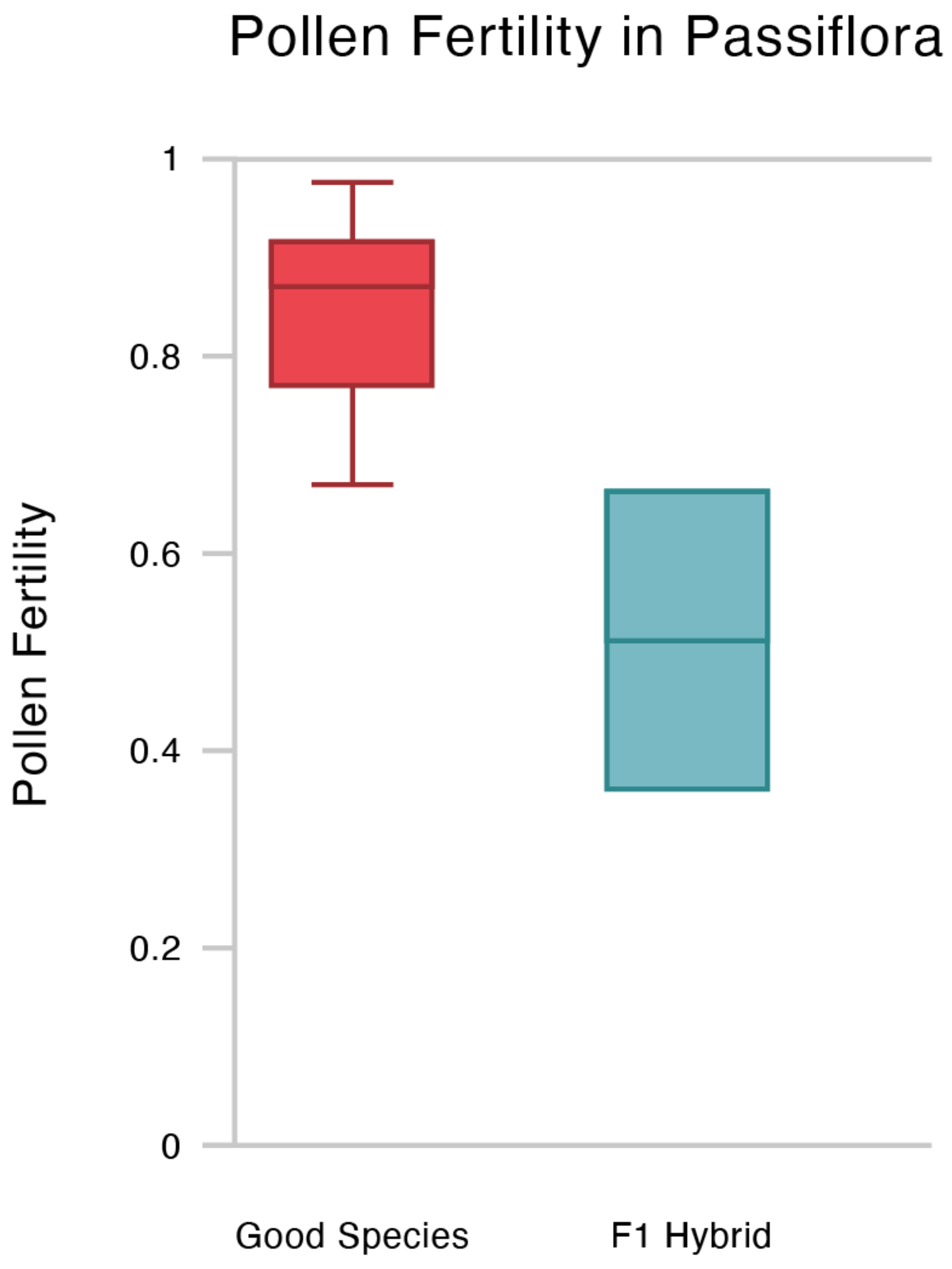

Supplementary Figure 5: *Passiflora* F1 hybrids (n=2) vs good species (n=11). The differences here are not statistically significant, probably due to sample size.

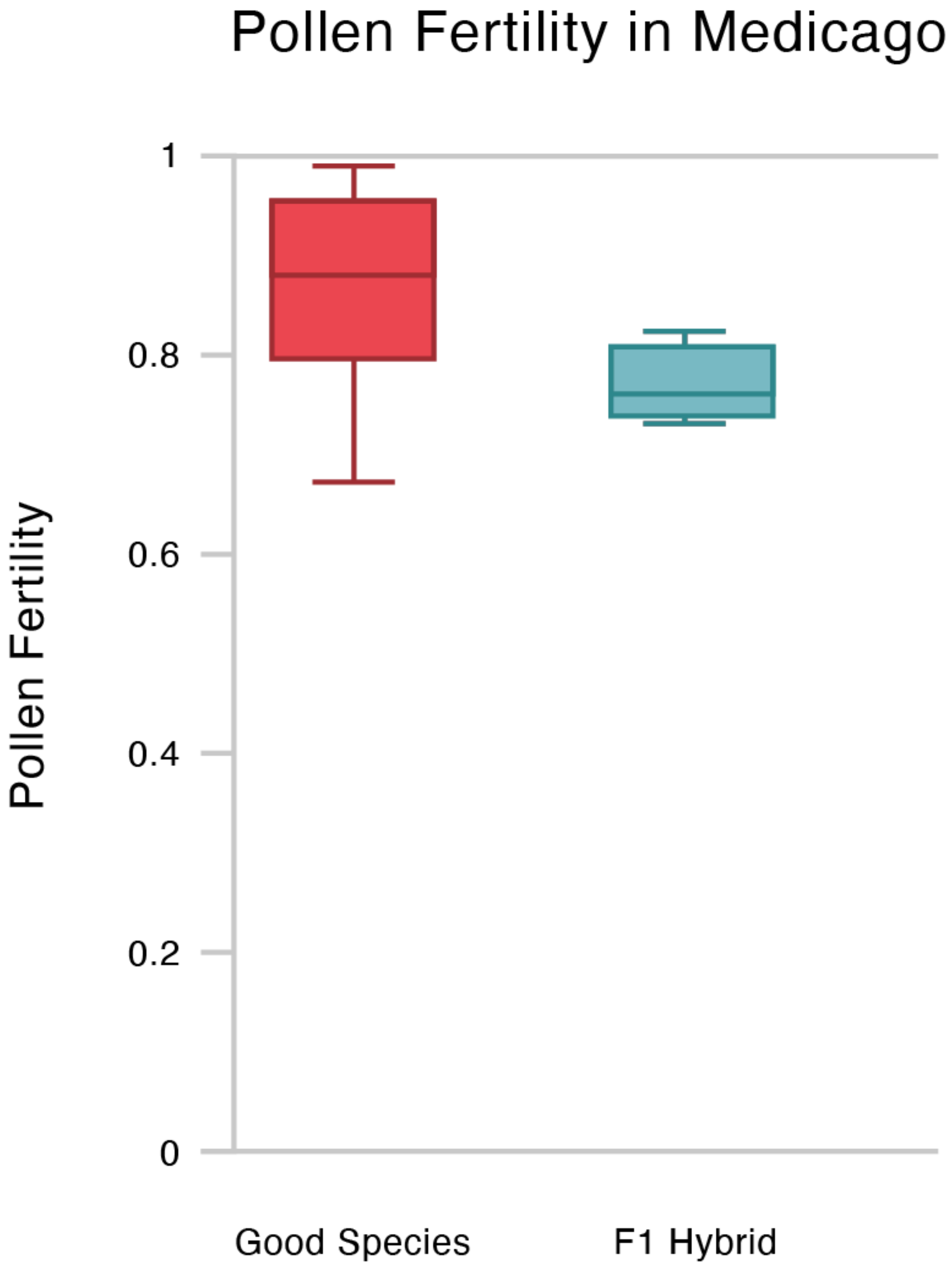

Supplementary Figure 6: *Medicago* F1 hybrids (n=3) vs good species (n=11). These differences are not significant

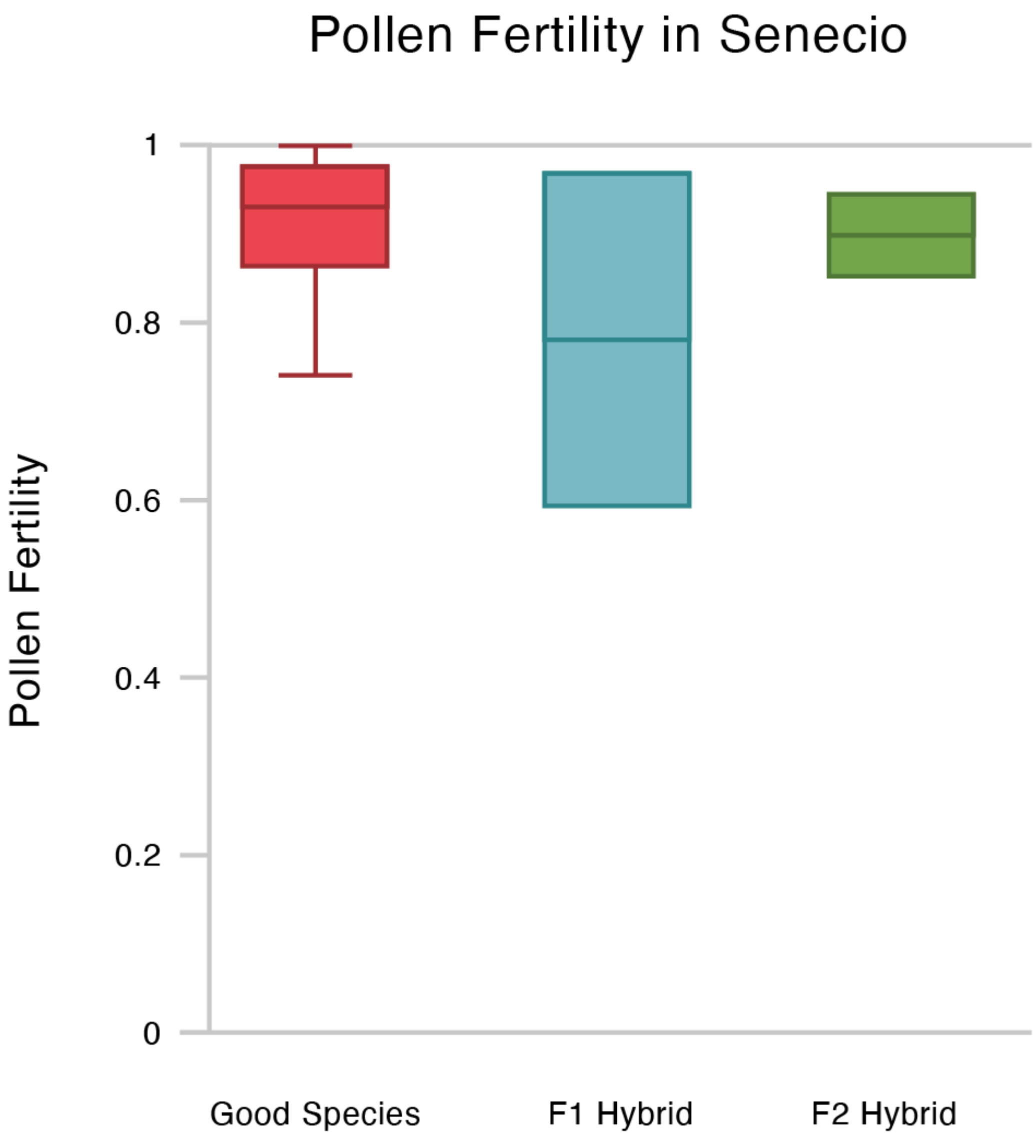

Supplementary Figure 7: *Senecio* F1 hybrids (n=2), F2 Hybrids (n=2), and good species (n=15).

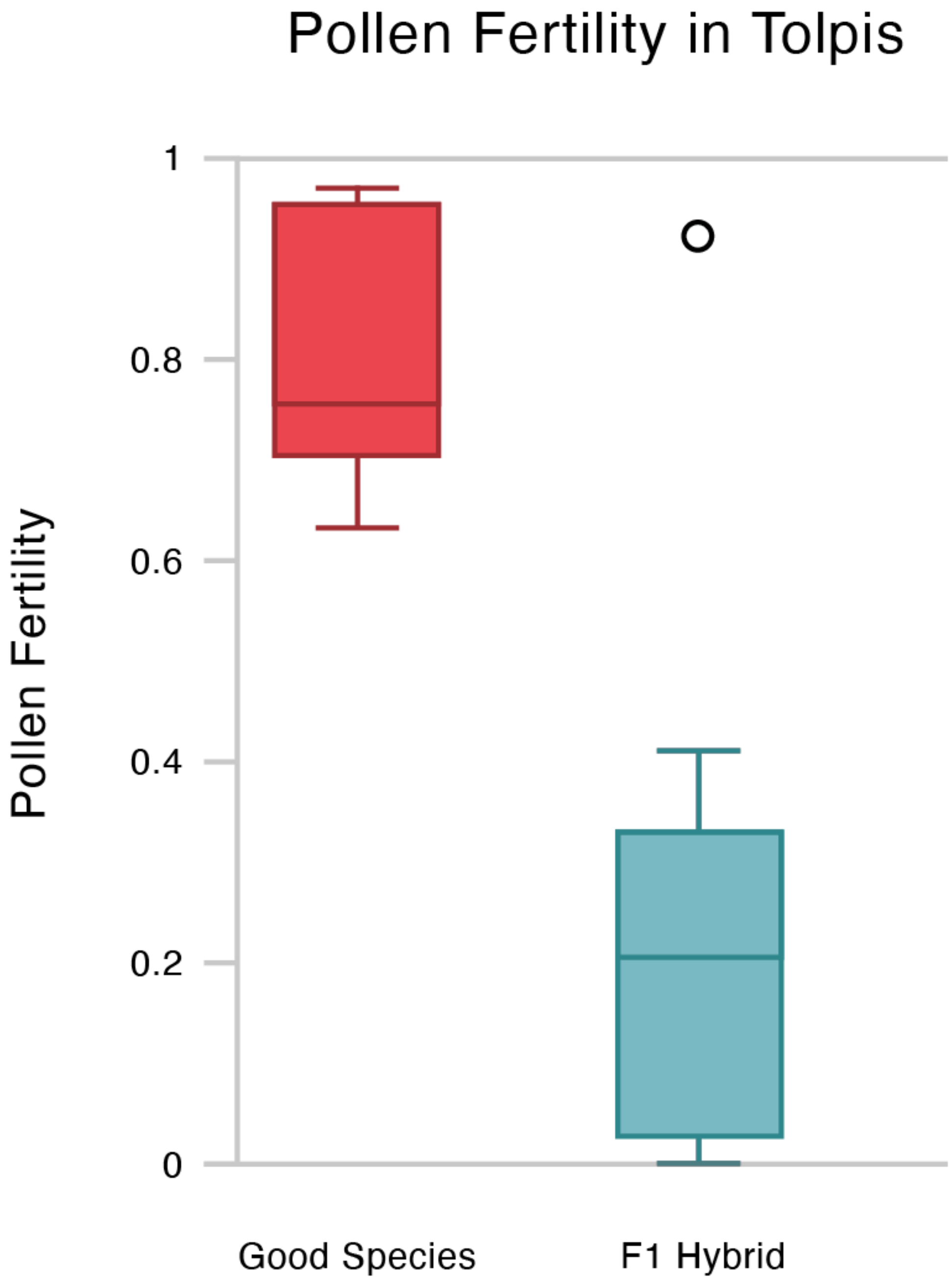

Supplementary Figure 8: *Tolpis* F1 hybrids (n=10) vs good species (n=8).

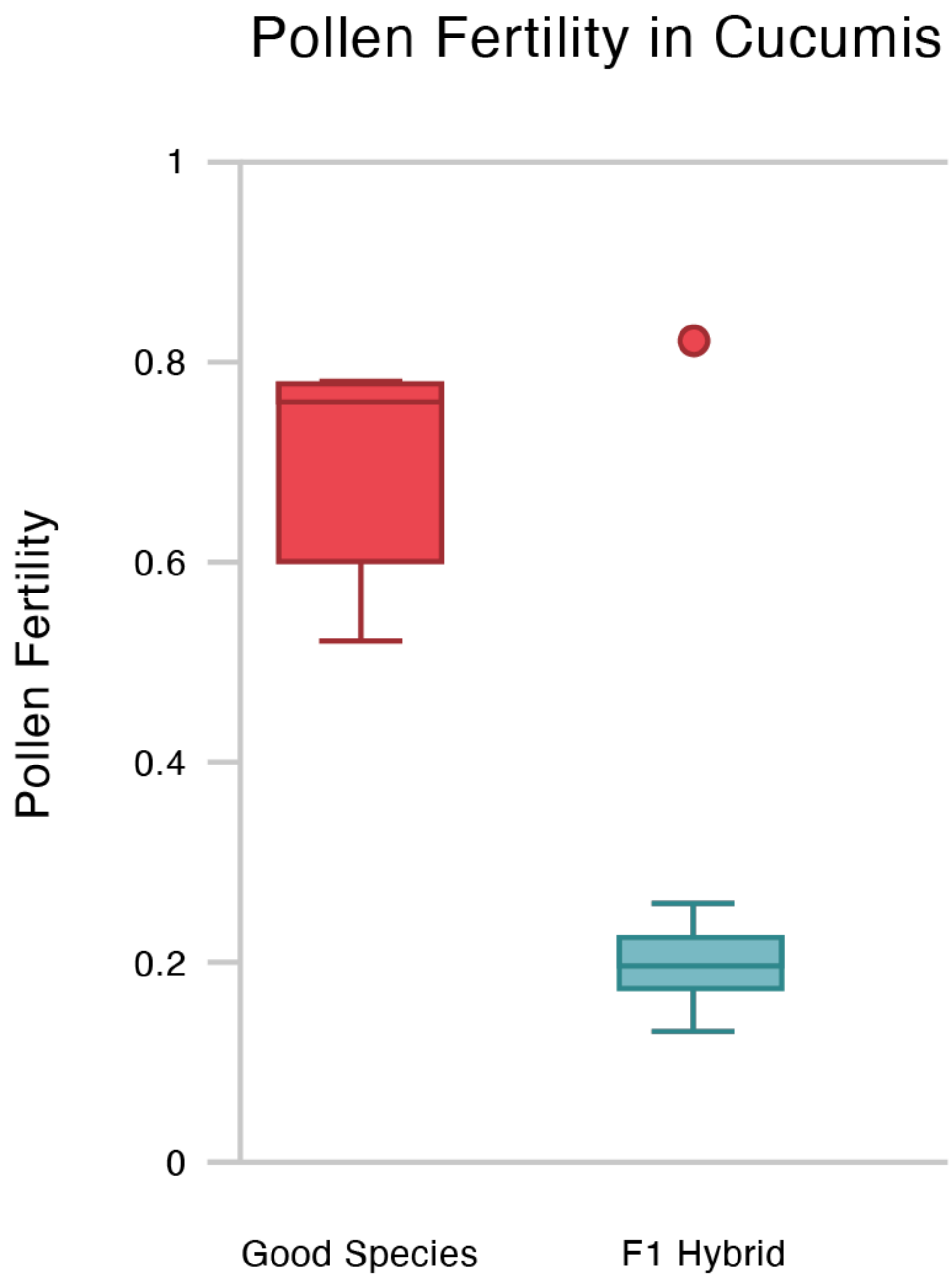

Supplementary Figure 9: *Cucumis* hybrids (n=11) vs good species (n=6).

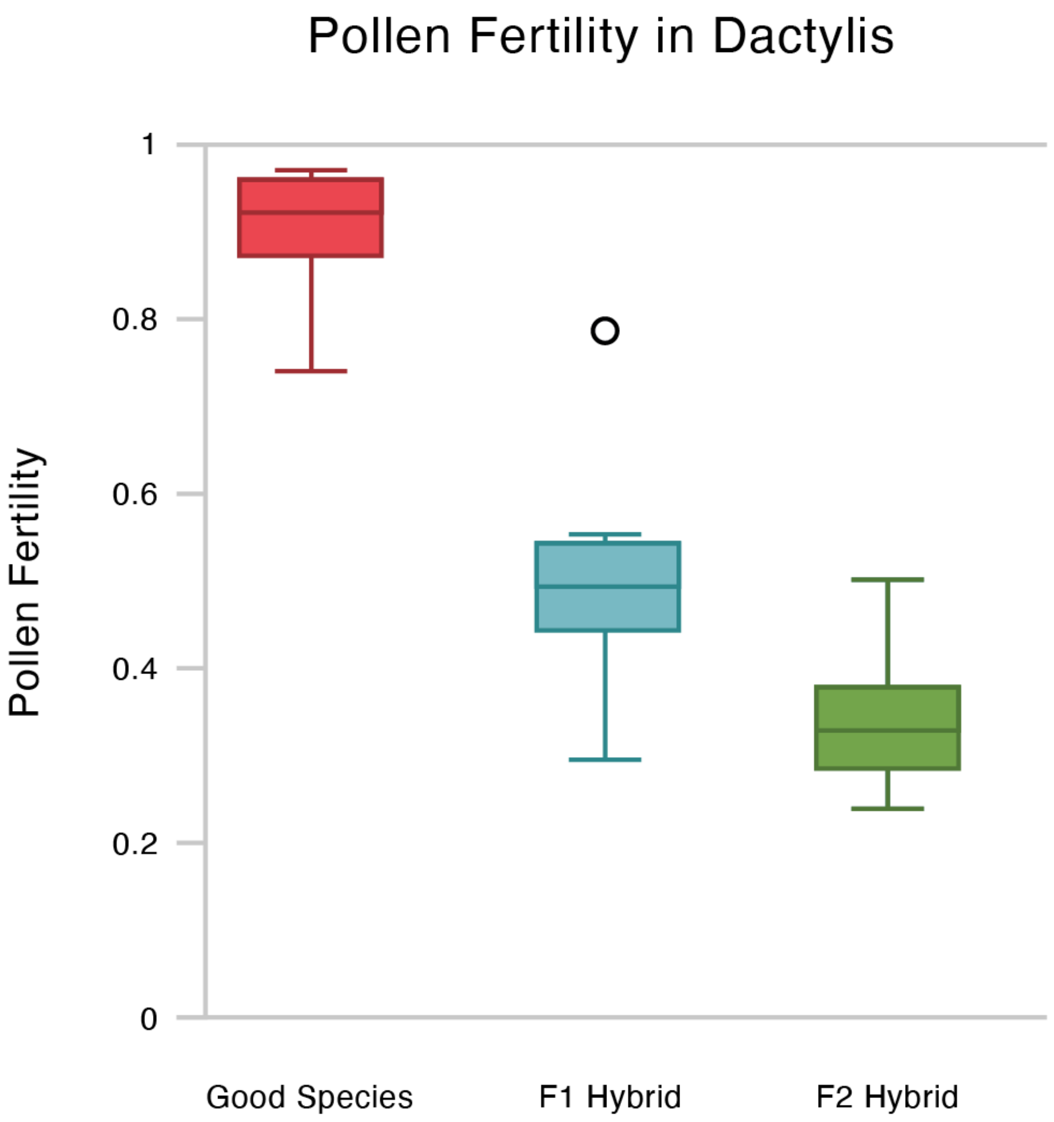

Supplementary Figure 10: *Dactylis* F1 hybrids (n=18), F2 hybrids (n=13) and good species (n=8). Note the drop between the F1 and F2 means.

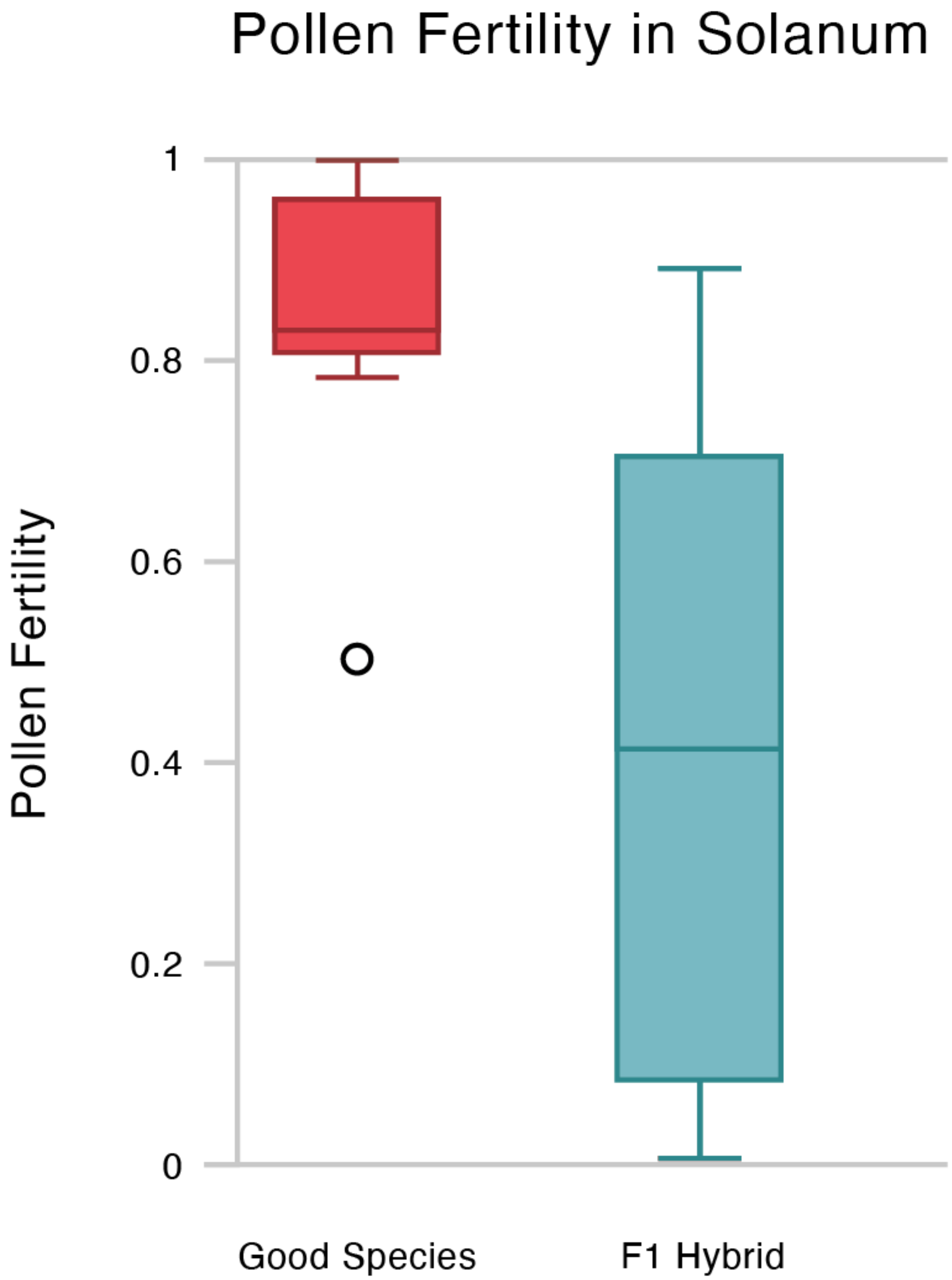

Supplementary Figure 11: Solanum F1 hybrids (n=7) vs good species (n=17).

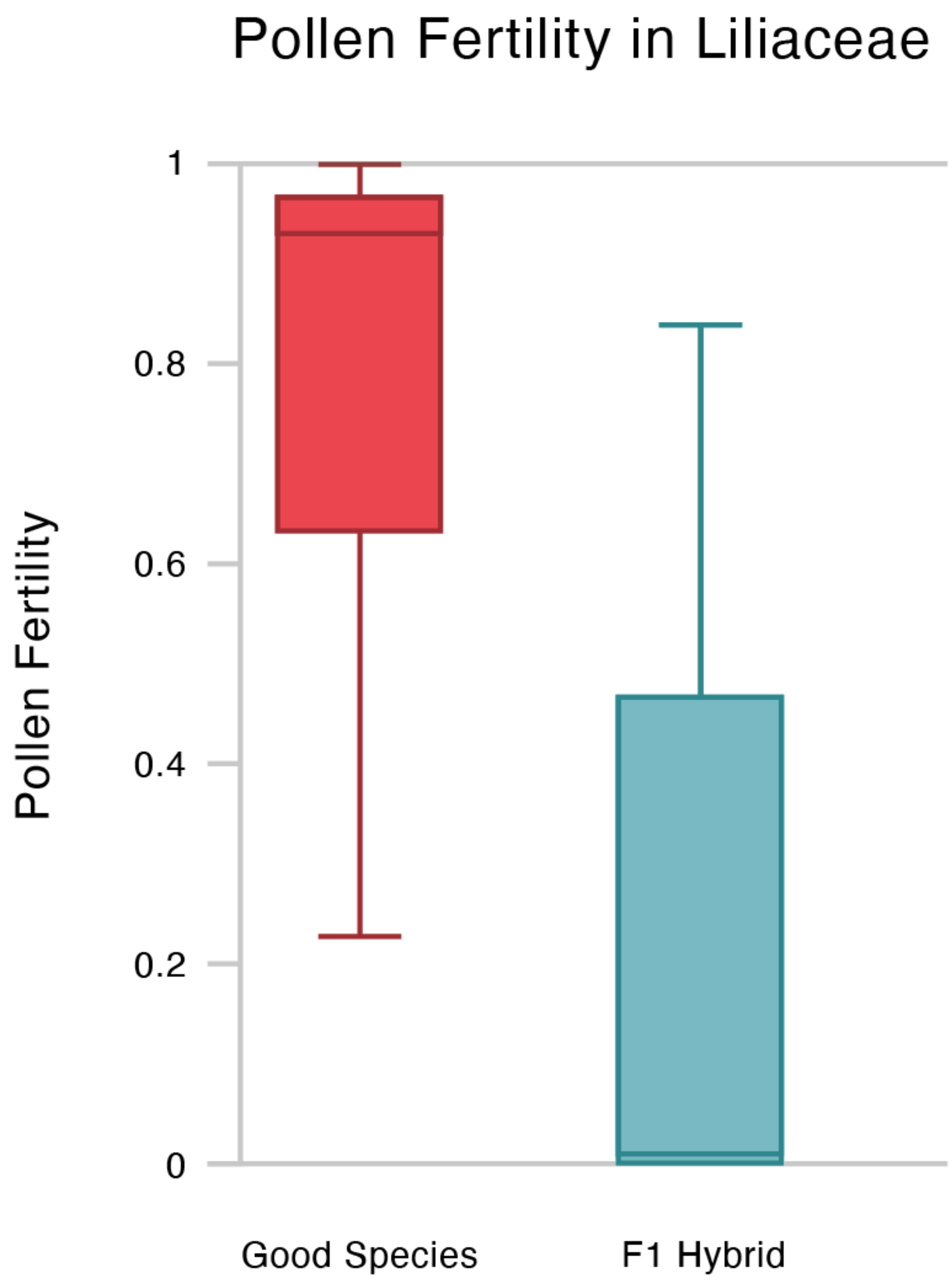

Supplementary Figure 11: Liliaceae F1 hybrids (n=8) vs good species (n=22).

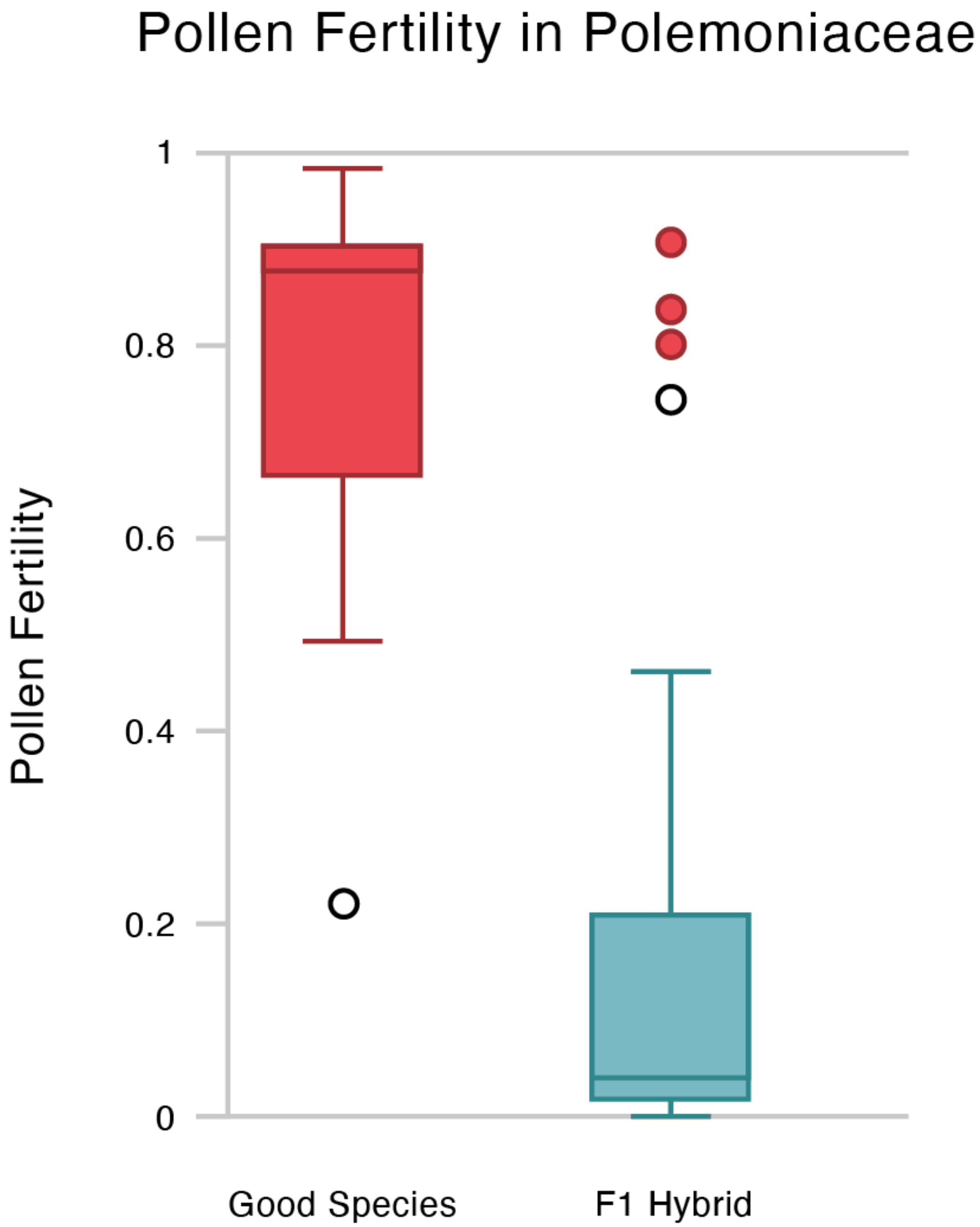

Supplementary Figure 12: Polemoniaceae F1 hybrids (n=22) vs good species (n=58). Note the variability in both groups. Potential outliers labeled in red.

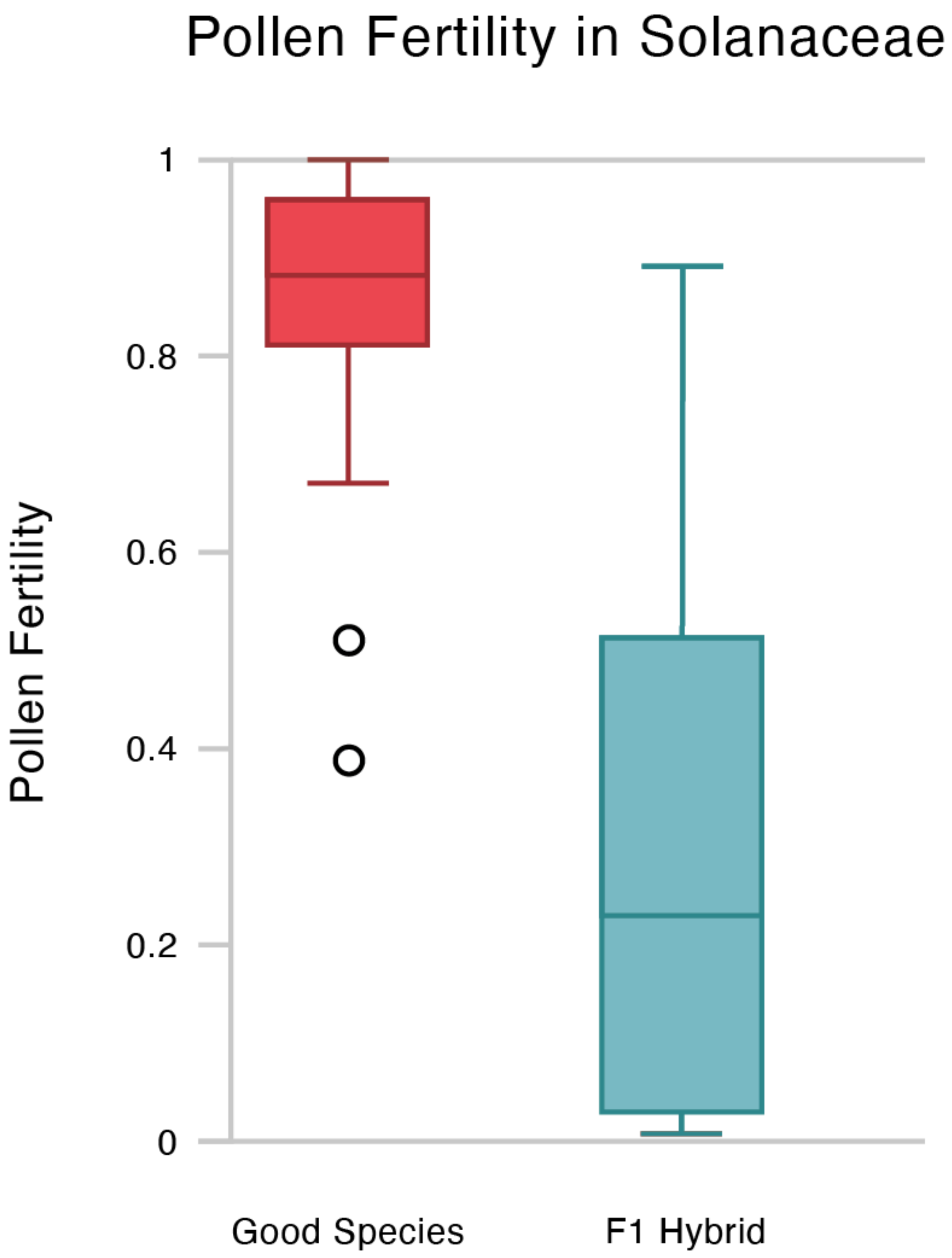

Supplementary Figure 13 Solanaceae F1 hybrids (n=16) vs good species (n=46).

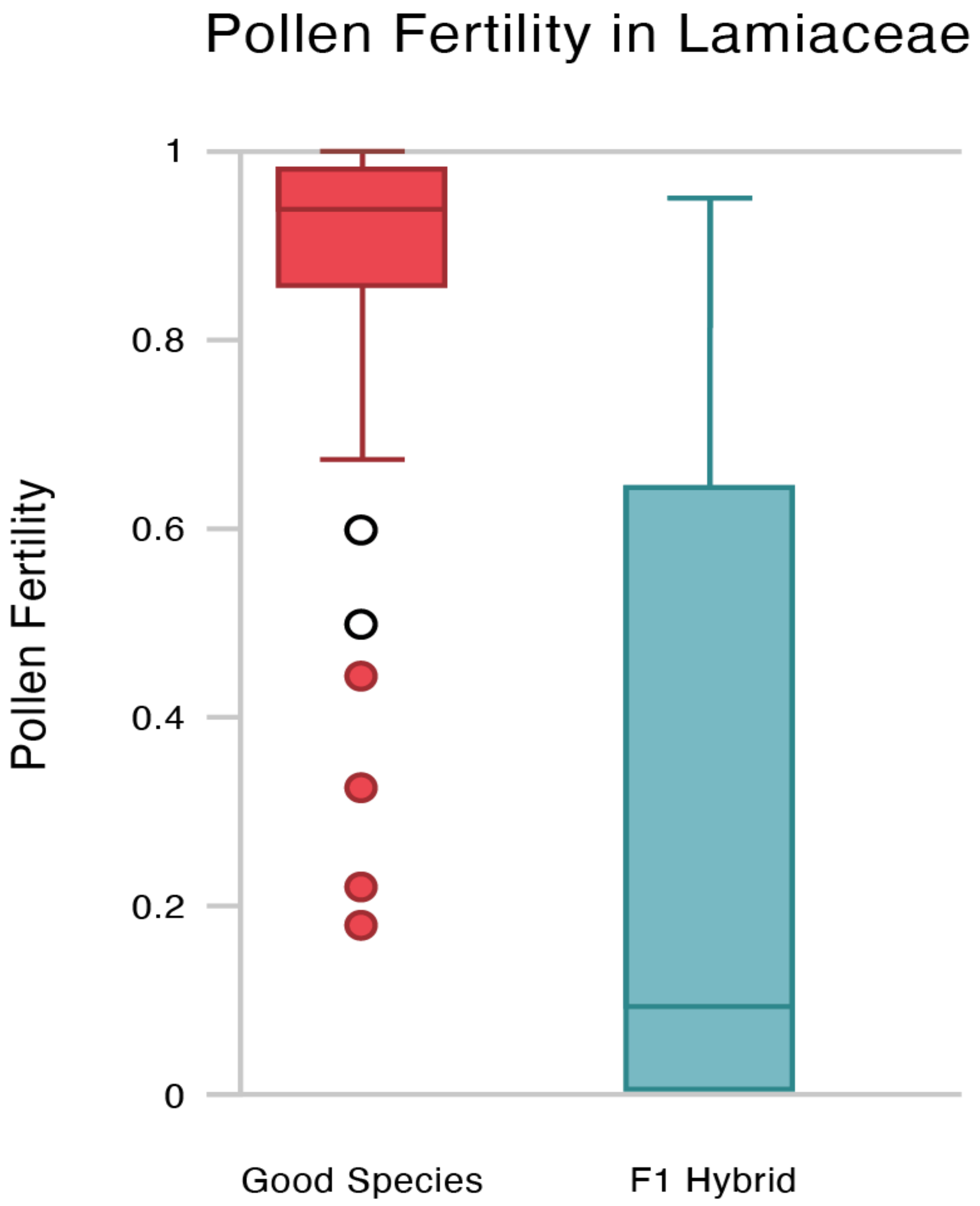

Supplementary Figure 14: Lamiaceae F1 hybrids (n=81) vs good species (n=11). While the results for this group are significant, there is great variability within both hybrids and good species. Potential outliers are labeled in red

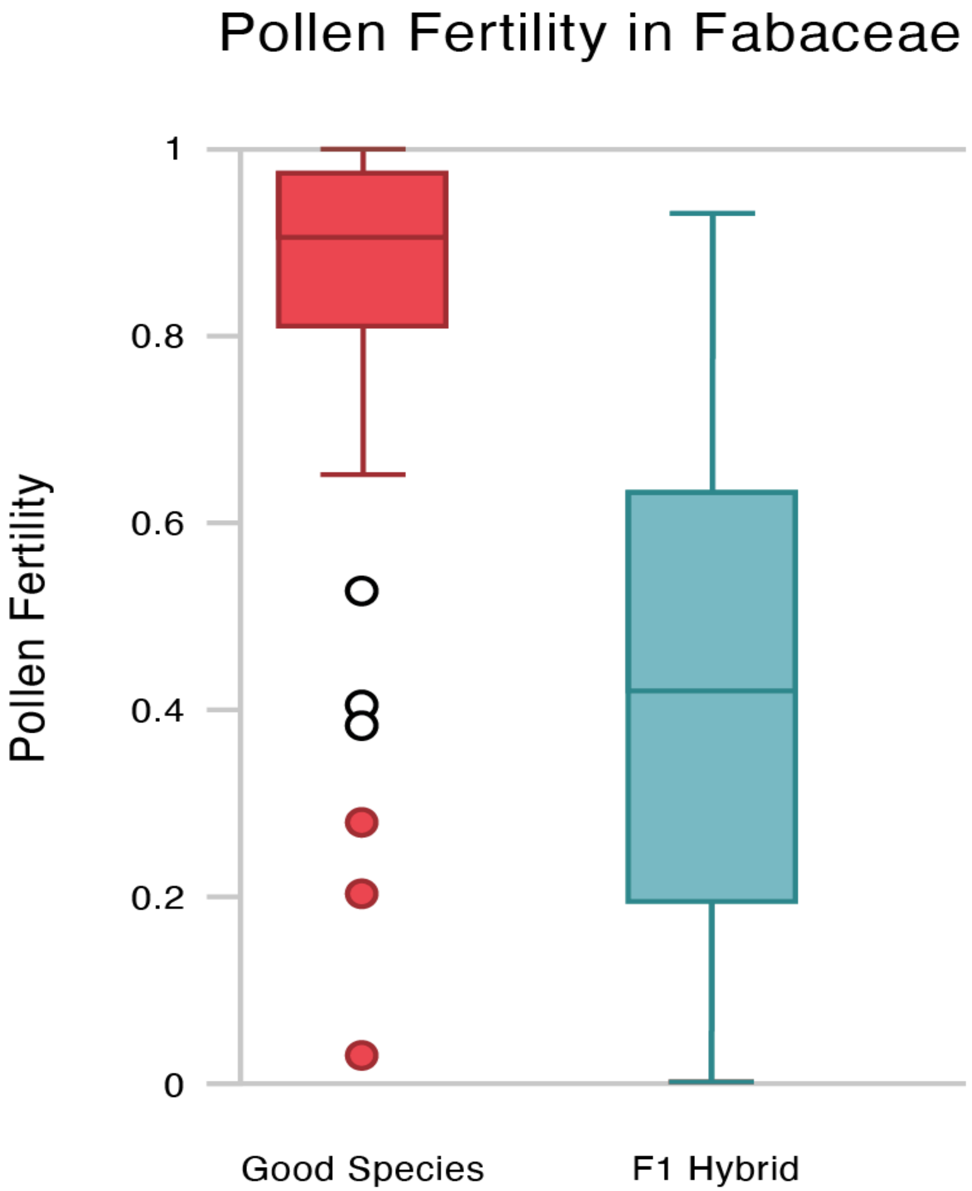

Supplementary Figure 15: Fabaceae F1 hybrids (n=28) vs good species (n=125). While the results for this group are significant, there is great variability within good species. Potential outliers labeled in red.

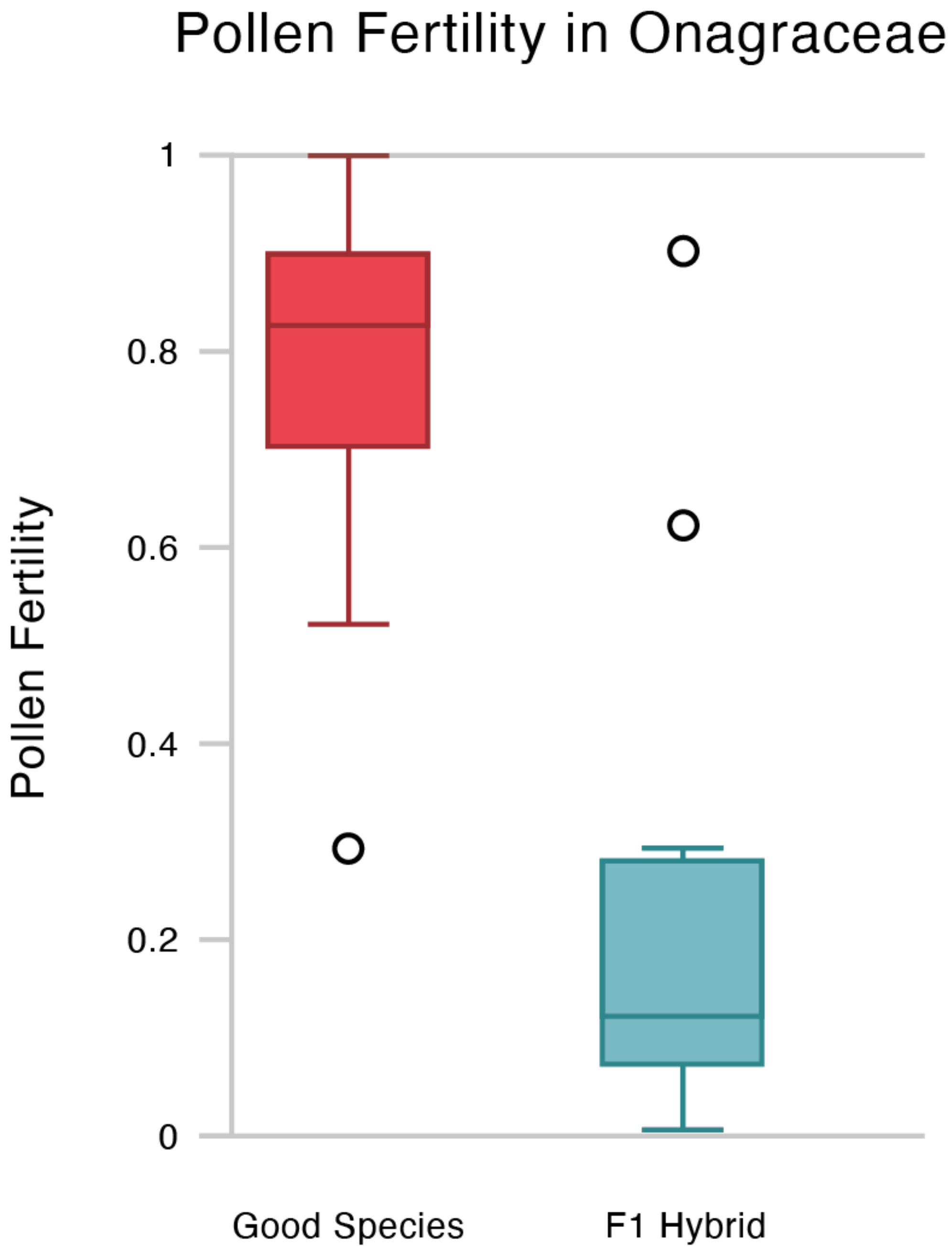

Supplementary Figure 16: Onagraceae F1 hybrids (n=11) vs good species (n=14).

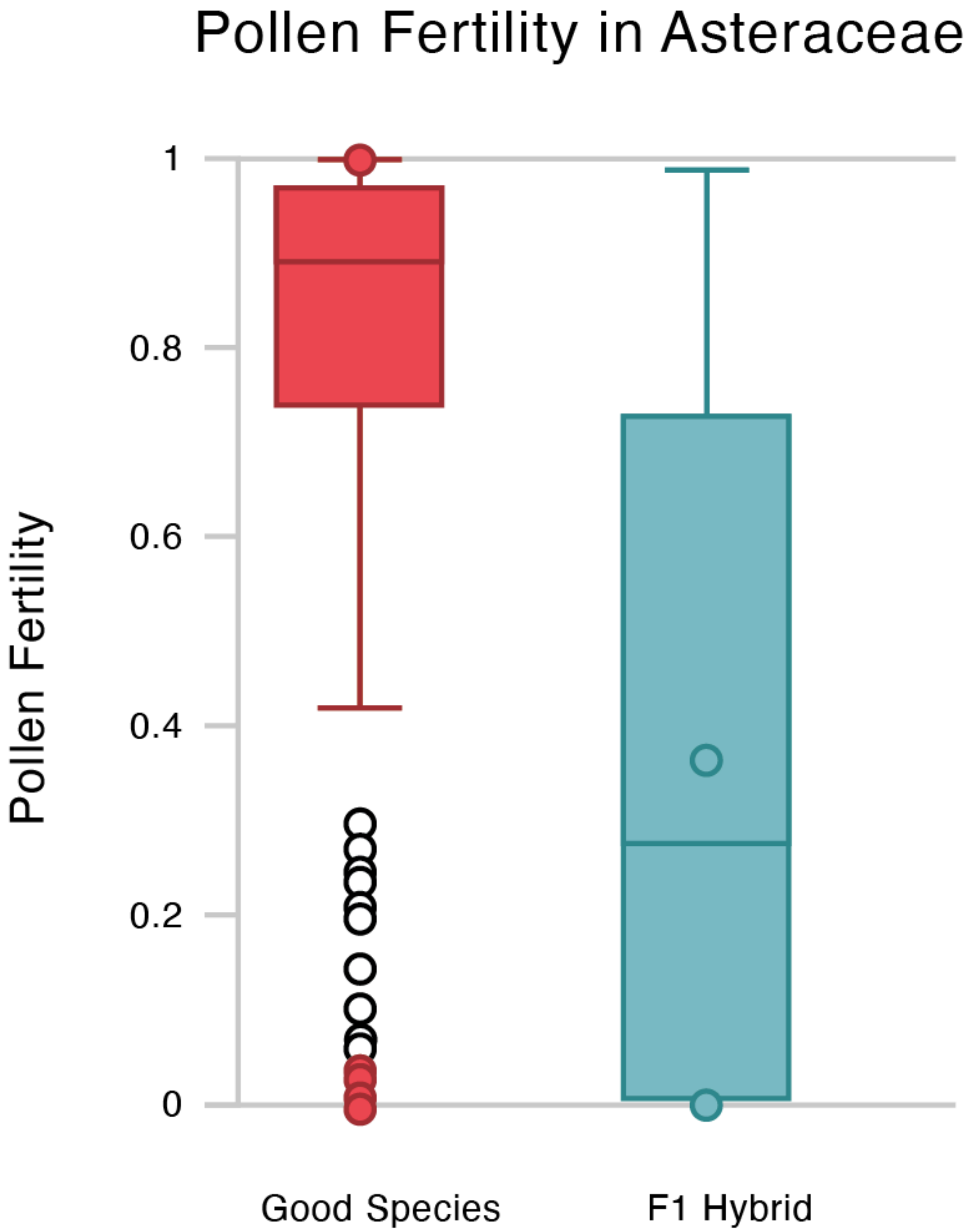

Supplementary Figure 17: Asteraceae F1 hybrids (Group 2) (n=178) vs good species (Group 1) (n=306). The large tail in the true species is composed mostly of the apomictic hawkweeds (*Hieracium*).

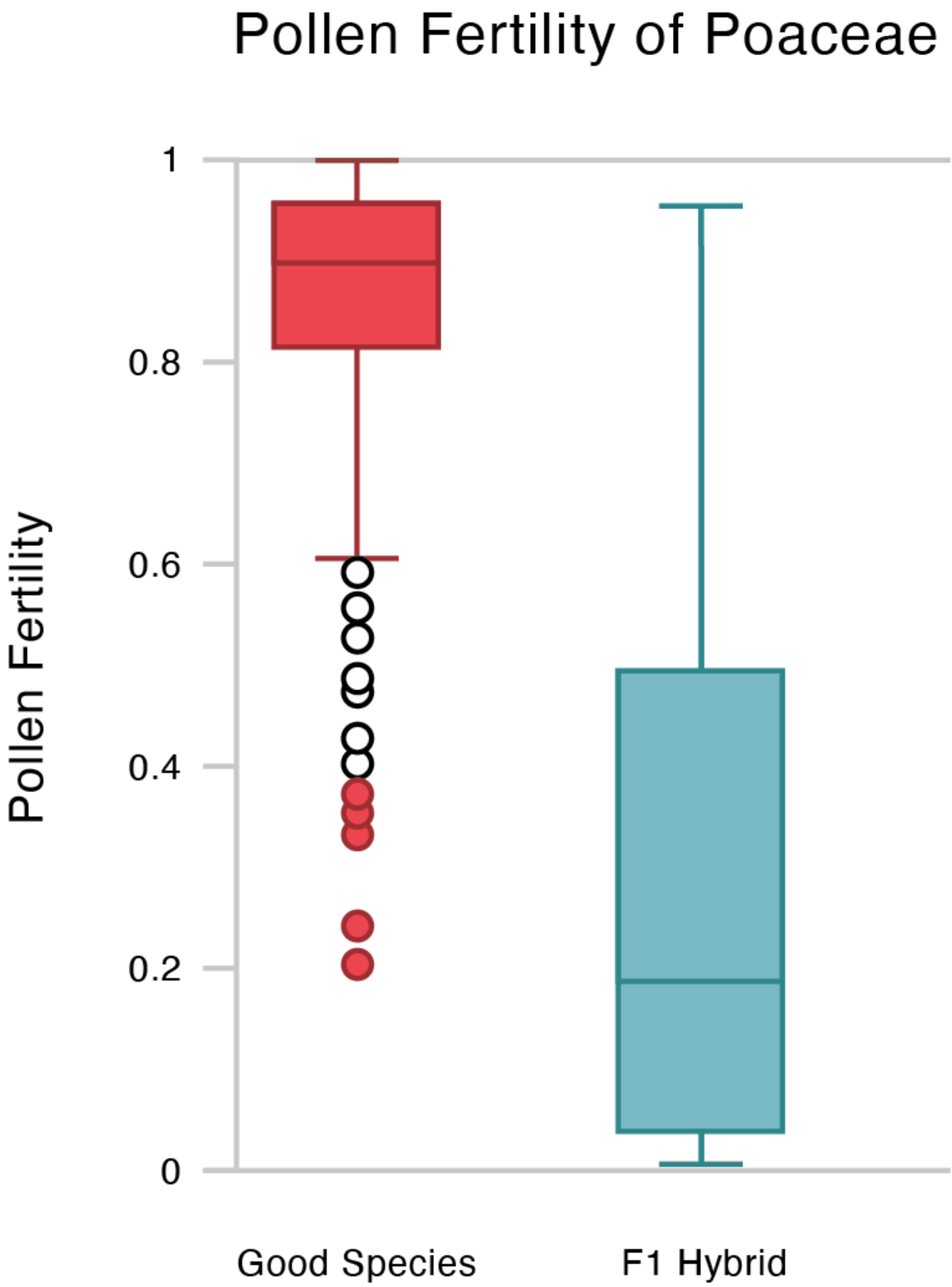

Supplementary Figure 18: Poaceae F1 hybrids (n=84) vs good species (n=208). Potential outliers are indicated in red.

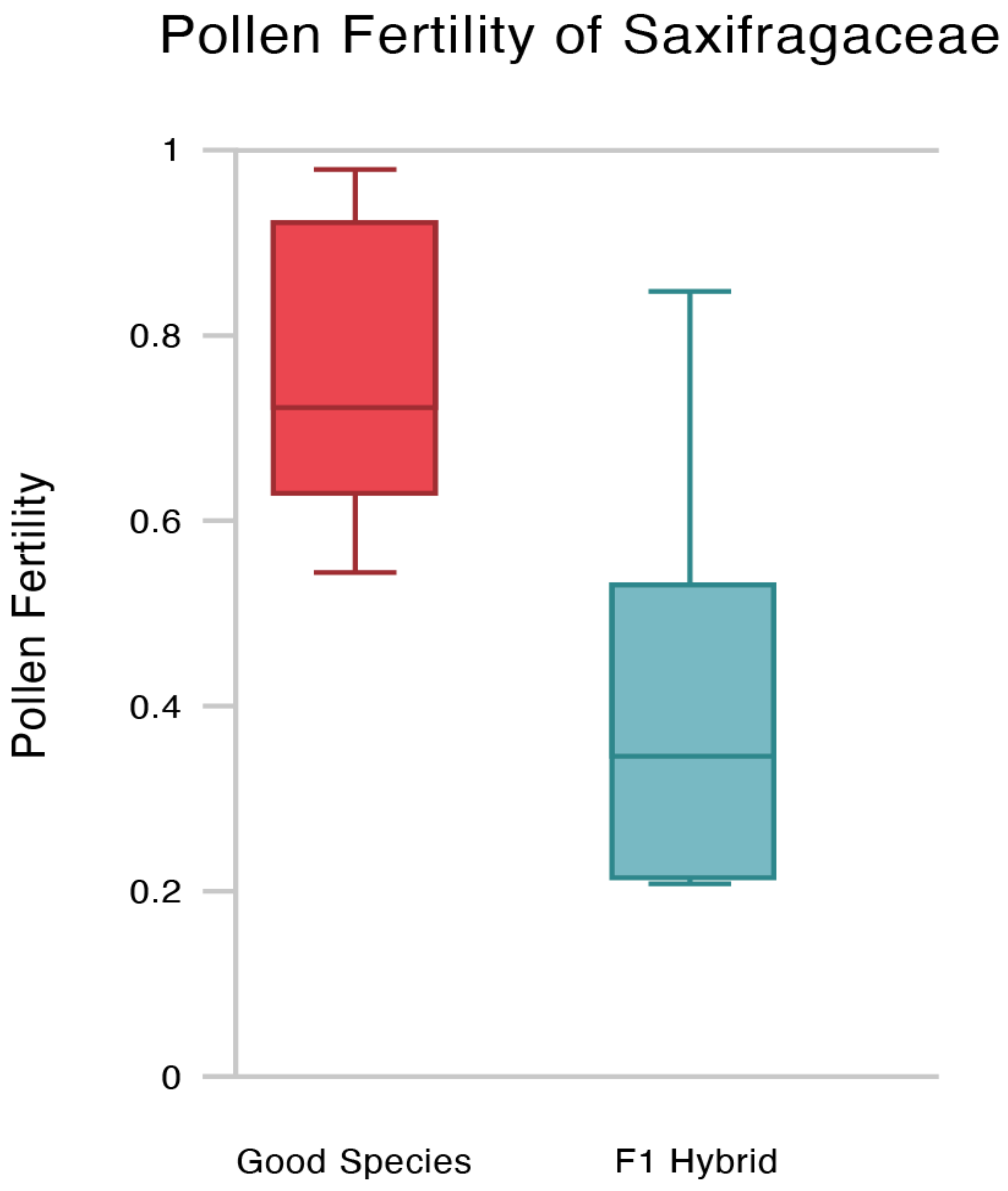

Supplementary Figure 19: Saxifragaceae F1 hybrids (n=5) vs good species (n=8).

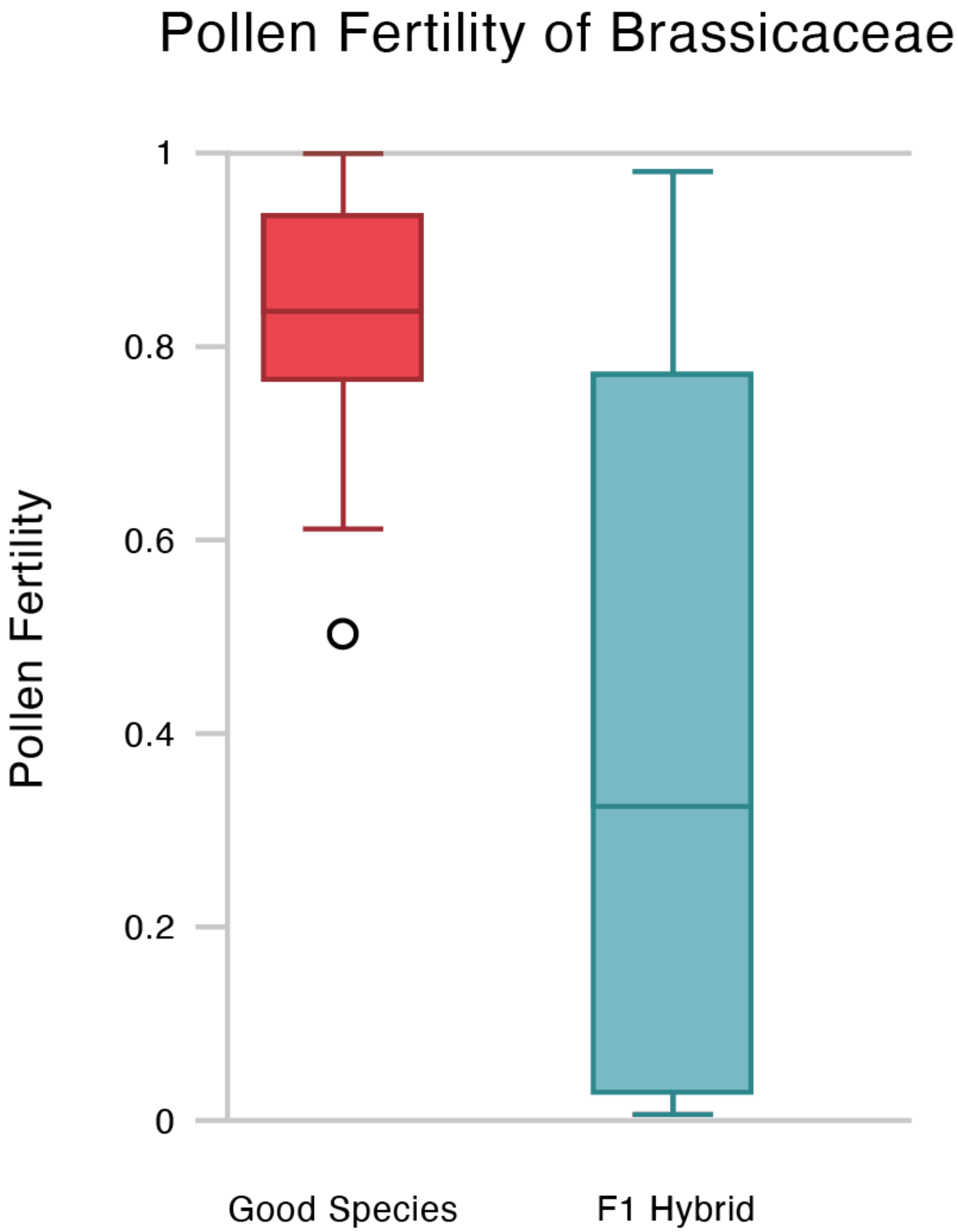

Supplementary Figure 20: Brassicaceae F1 hybrids (n=31) vs good species (n=38). Note the wide range of variability in the hybrids.

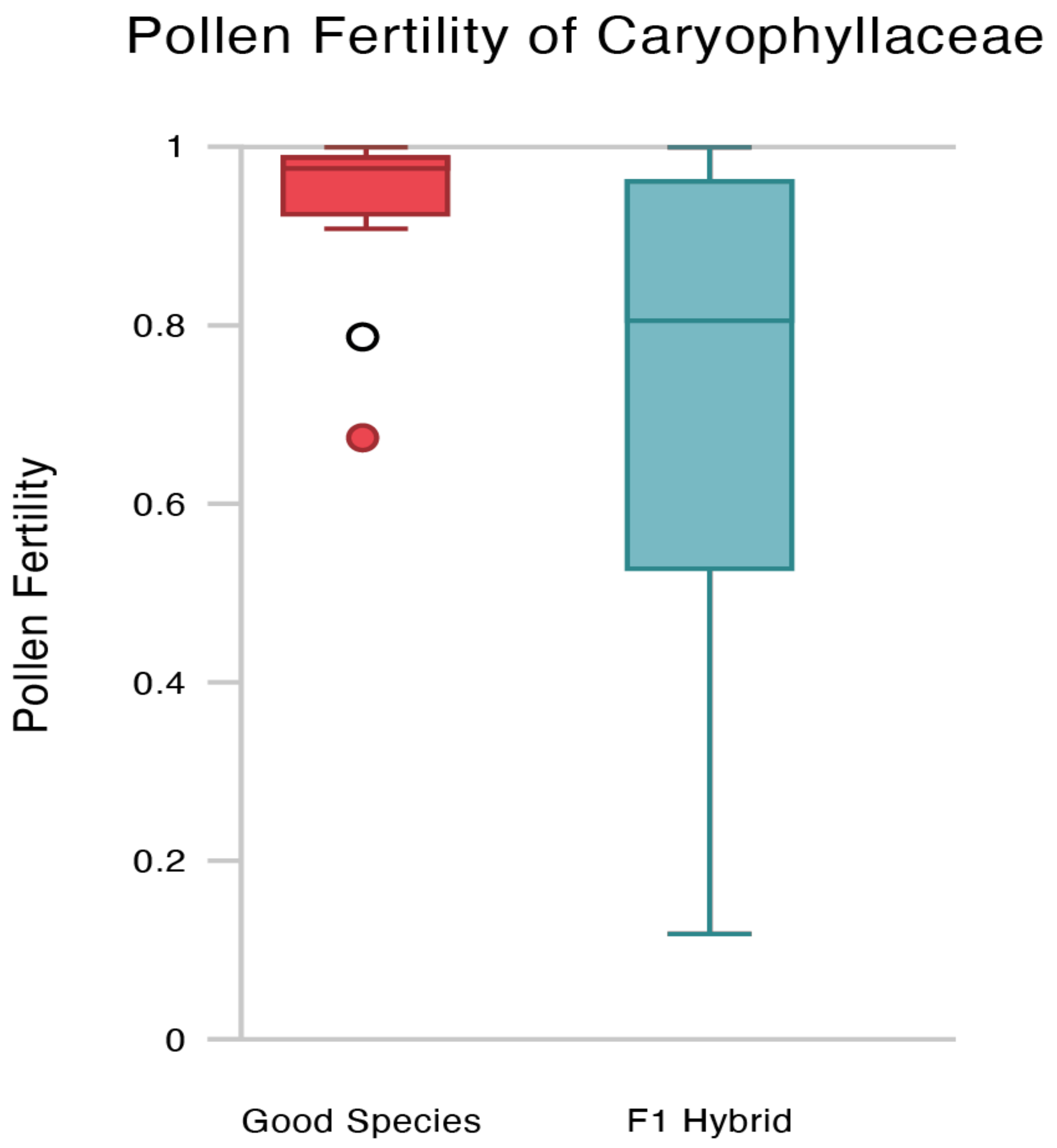

Supplementary Figure 21: Caryophyllaceae F1 hybrids (n=8) vs good species (n=24). Results for this group are non-significant, though hybrids have a broader range. Potential outliers are highlighted in red.

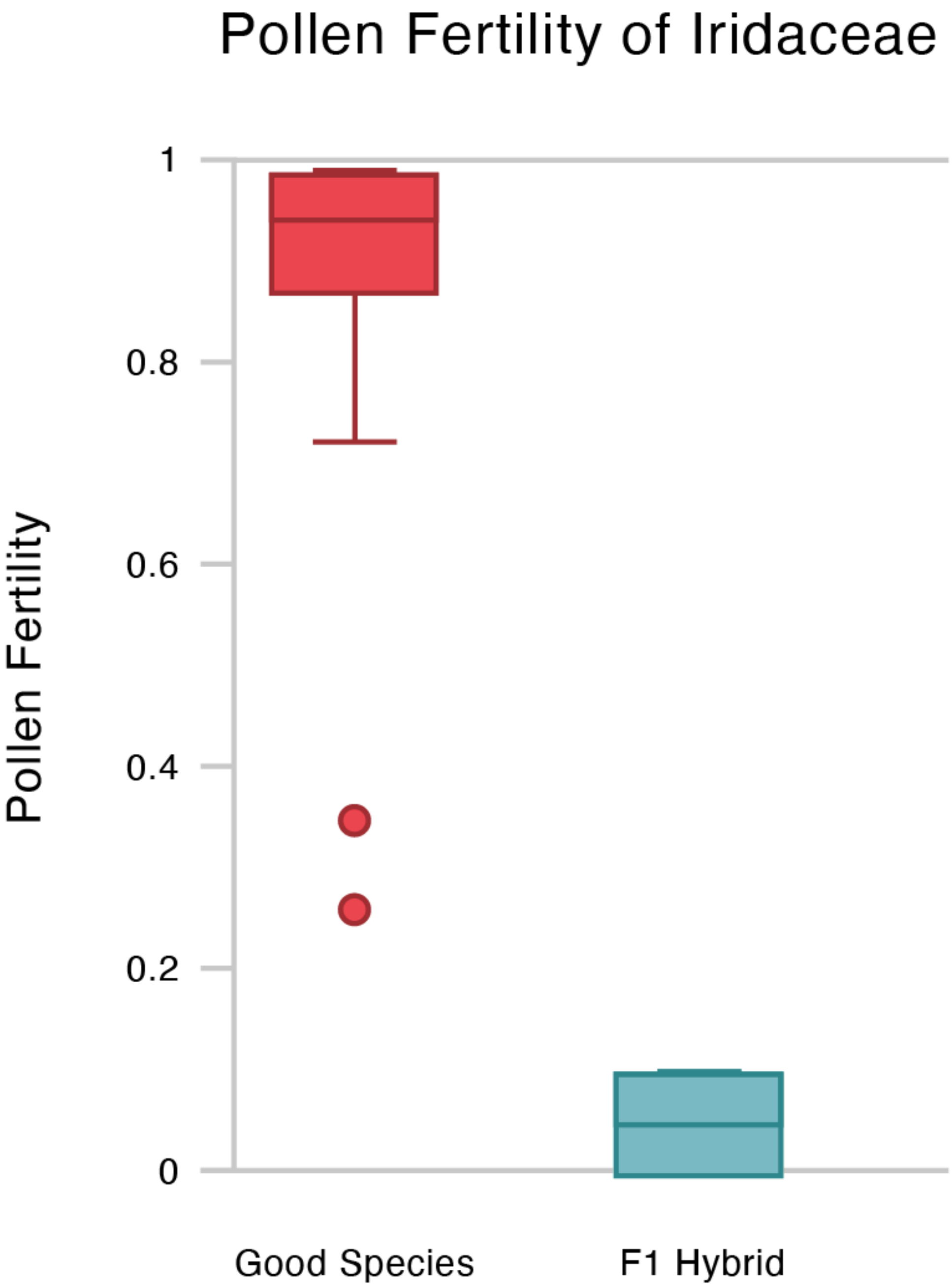

Supplementary Figure 22: Iridaceae F1 hybrids (n=2) vs good species (n=20). Potential outliers shown in red.

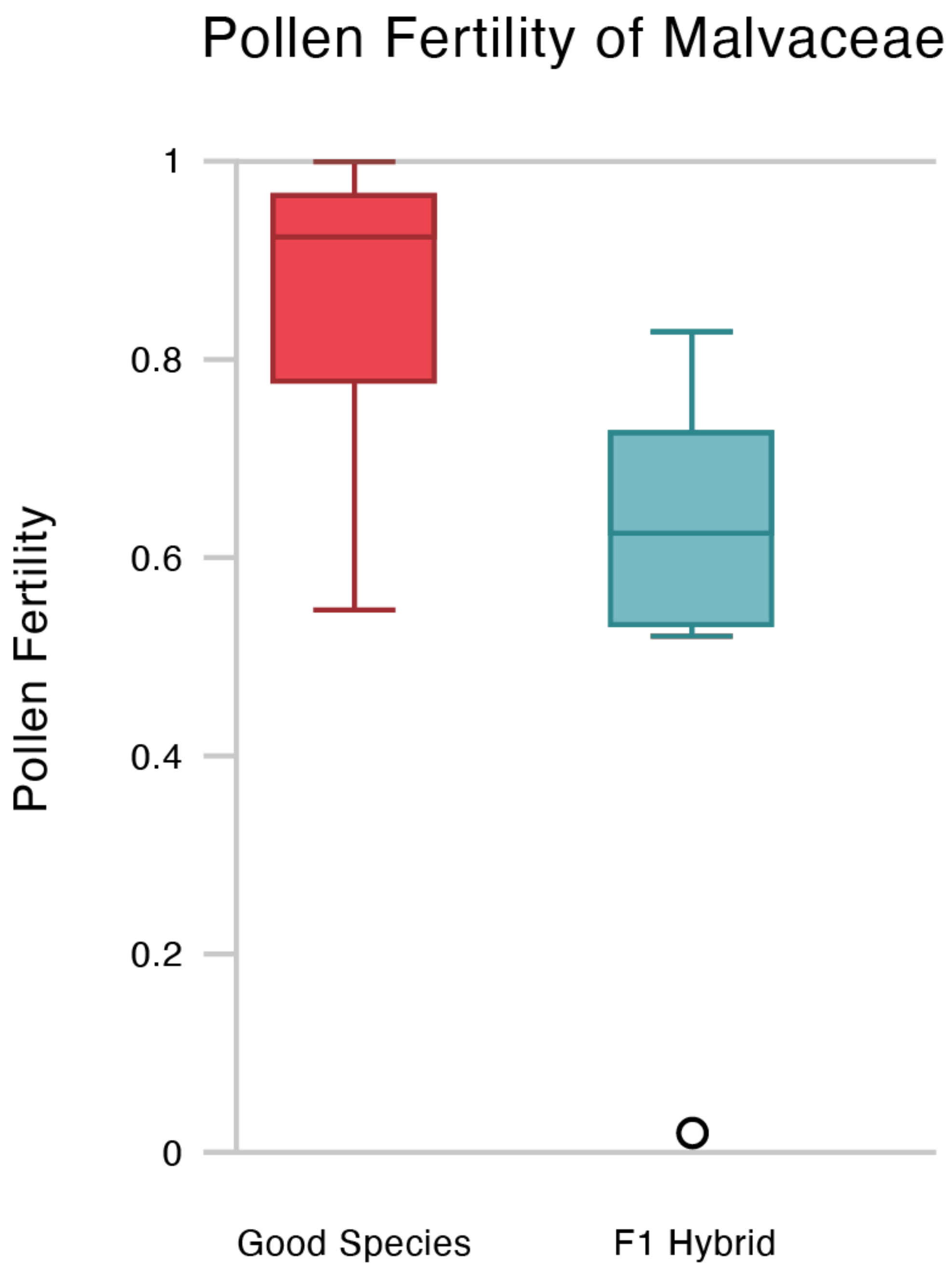

Supplementary Figure 23: Malvaceae F1 hybrids (n=7) vs good species (n=25).

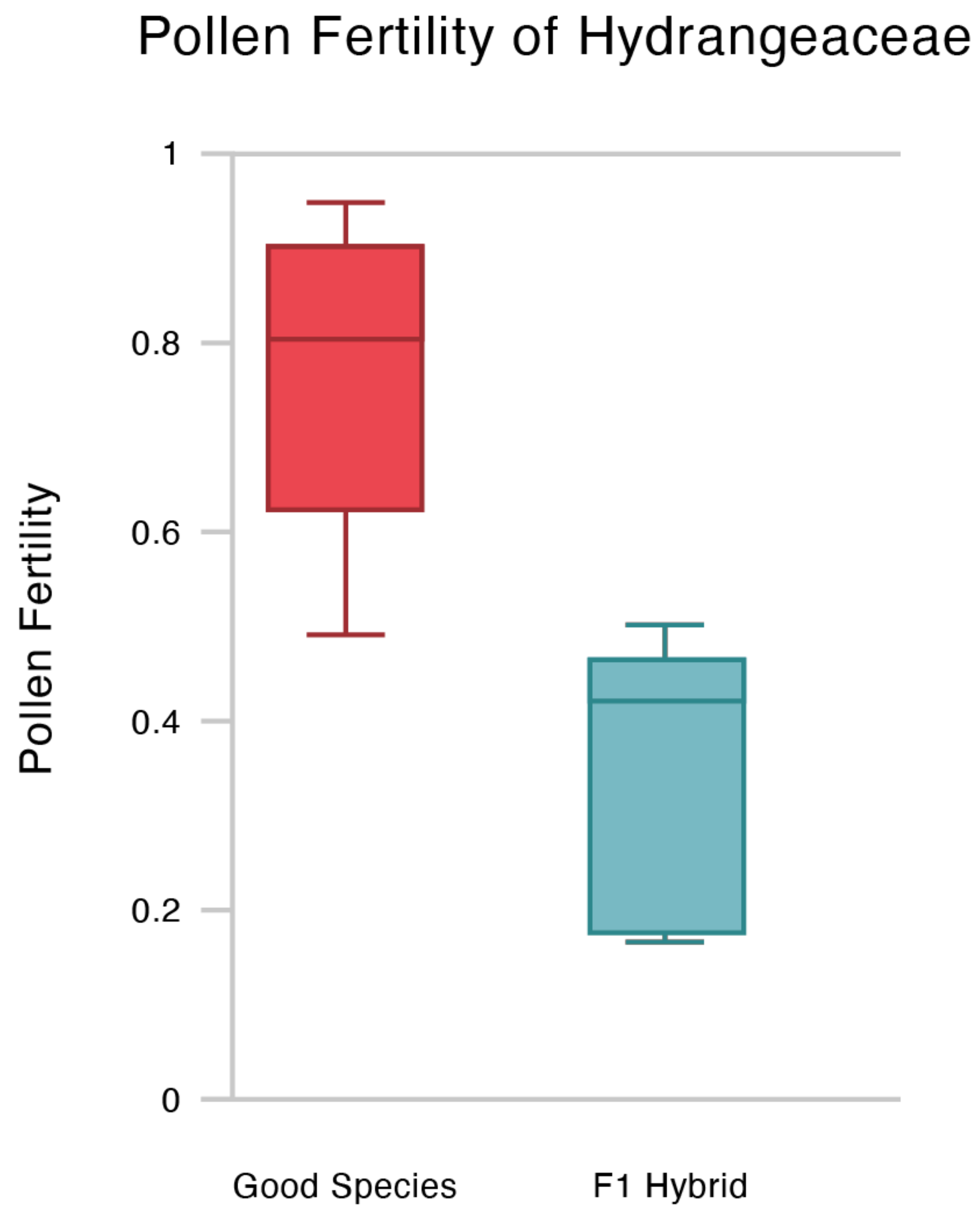

Supplementary Figure 24: Hydrangeaceae F1 hybrids (n=5) vs good species (n=16).

.

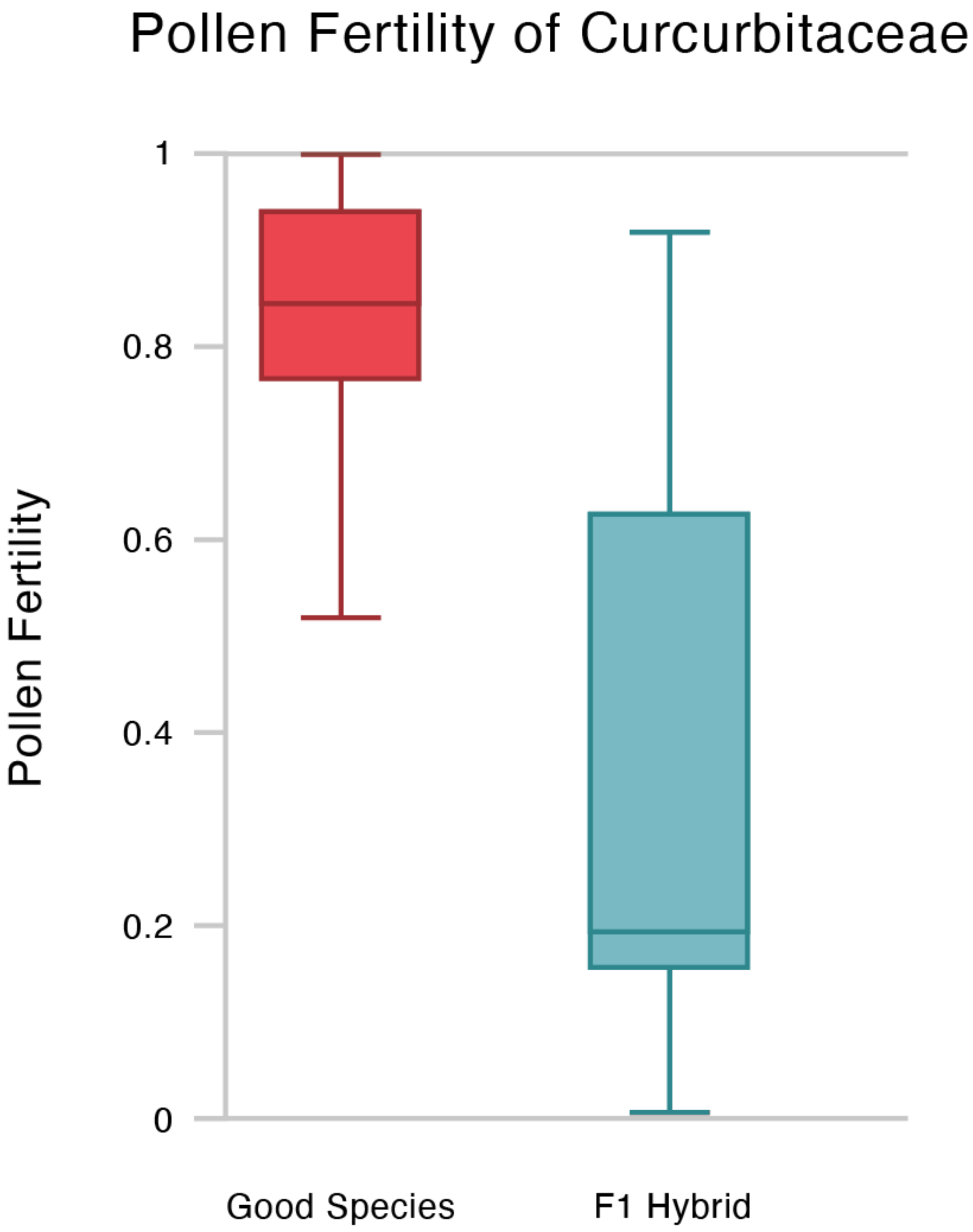

Supplementary Figure 25: Cucurbitaceae F1 hybrids (n=21) vs true species (n=14).

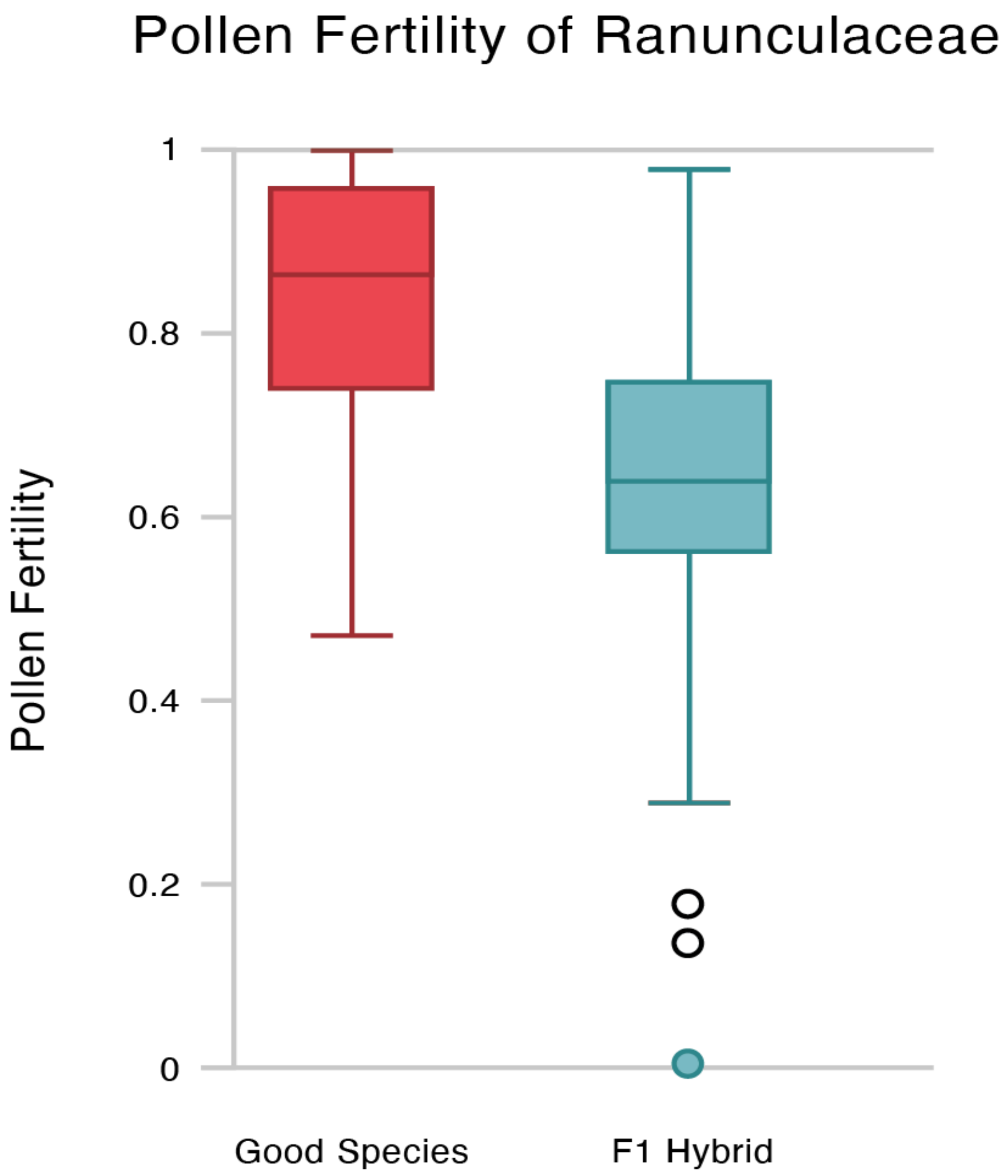

Supplementary Figure 26: Ranunculaceae F1 hybrids (n=66) vs good species (n=28). Potential outliers are highlighted in red. This is one of the few groups with more hybrids than good species.

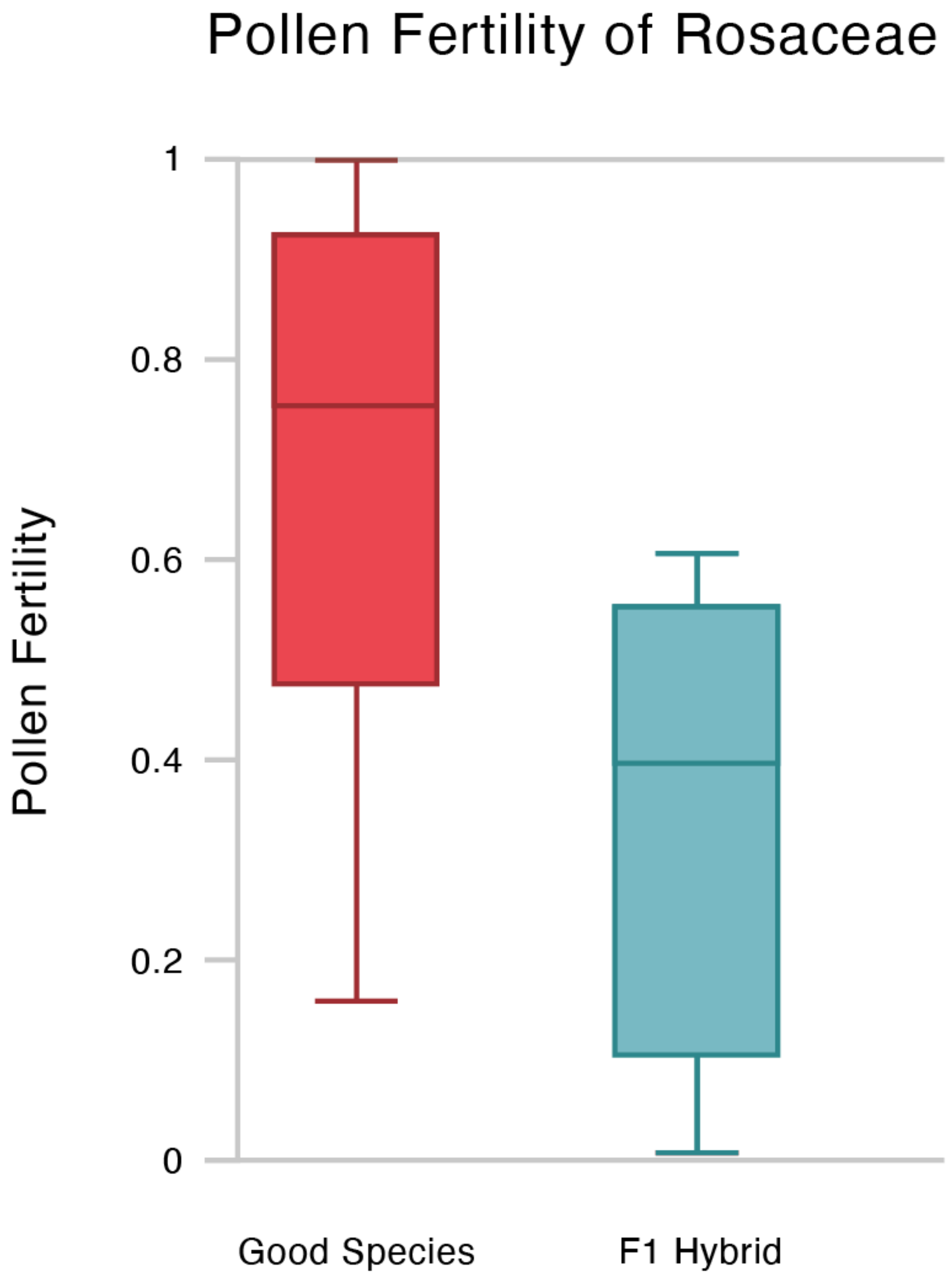

Supplementary Figure 27: Rosaceae F1 hybrids (n=3) vs good species (n=50). Results for this group are non-significant likely due to the low sample size of F1s.

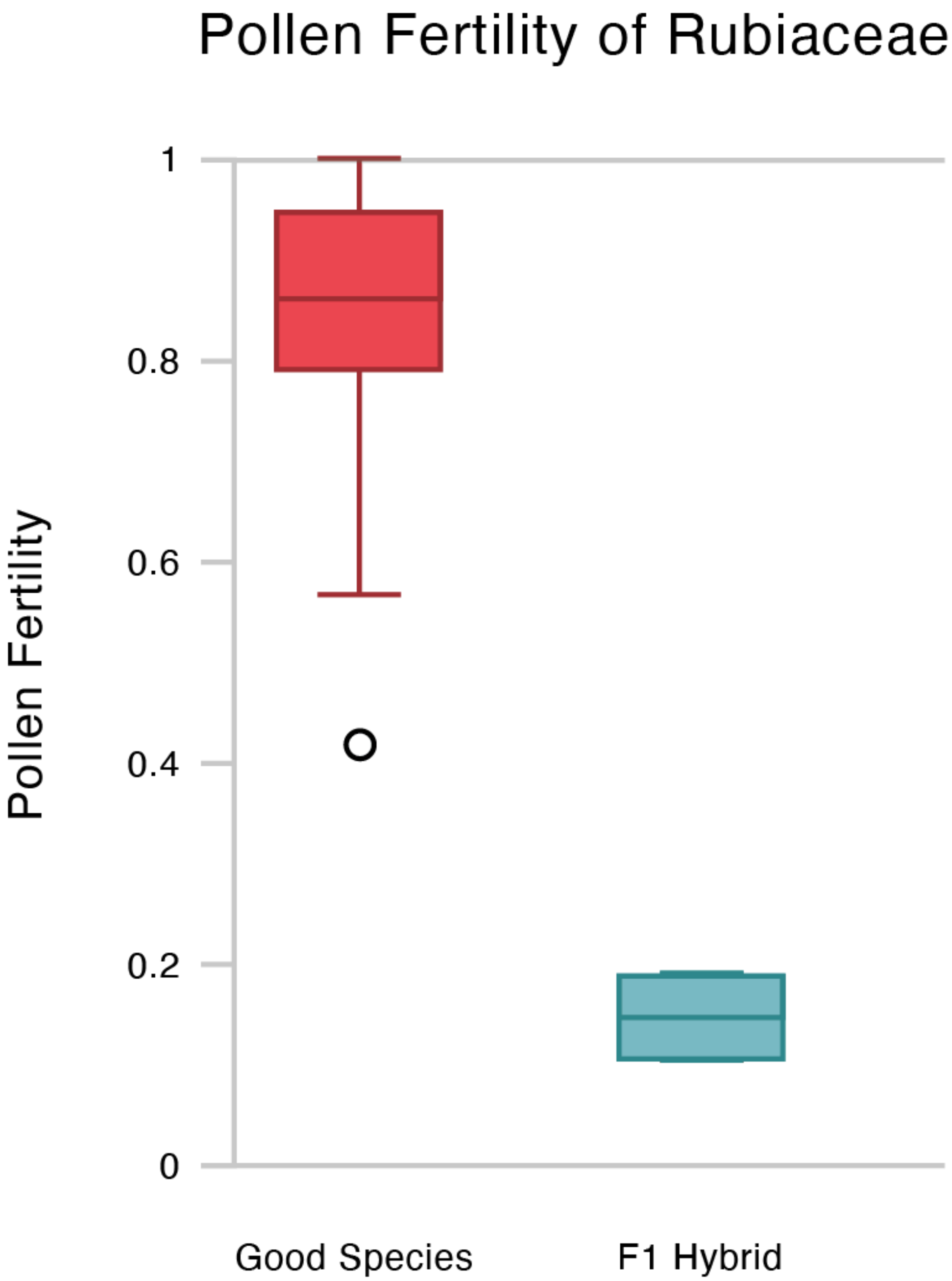

Supplementary Figure 28: Rubiaceae F1 hybrids (n=2) vs good species (n=34).

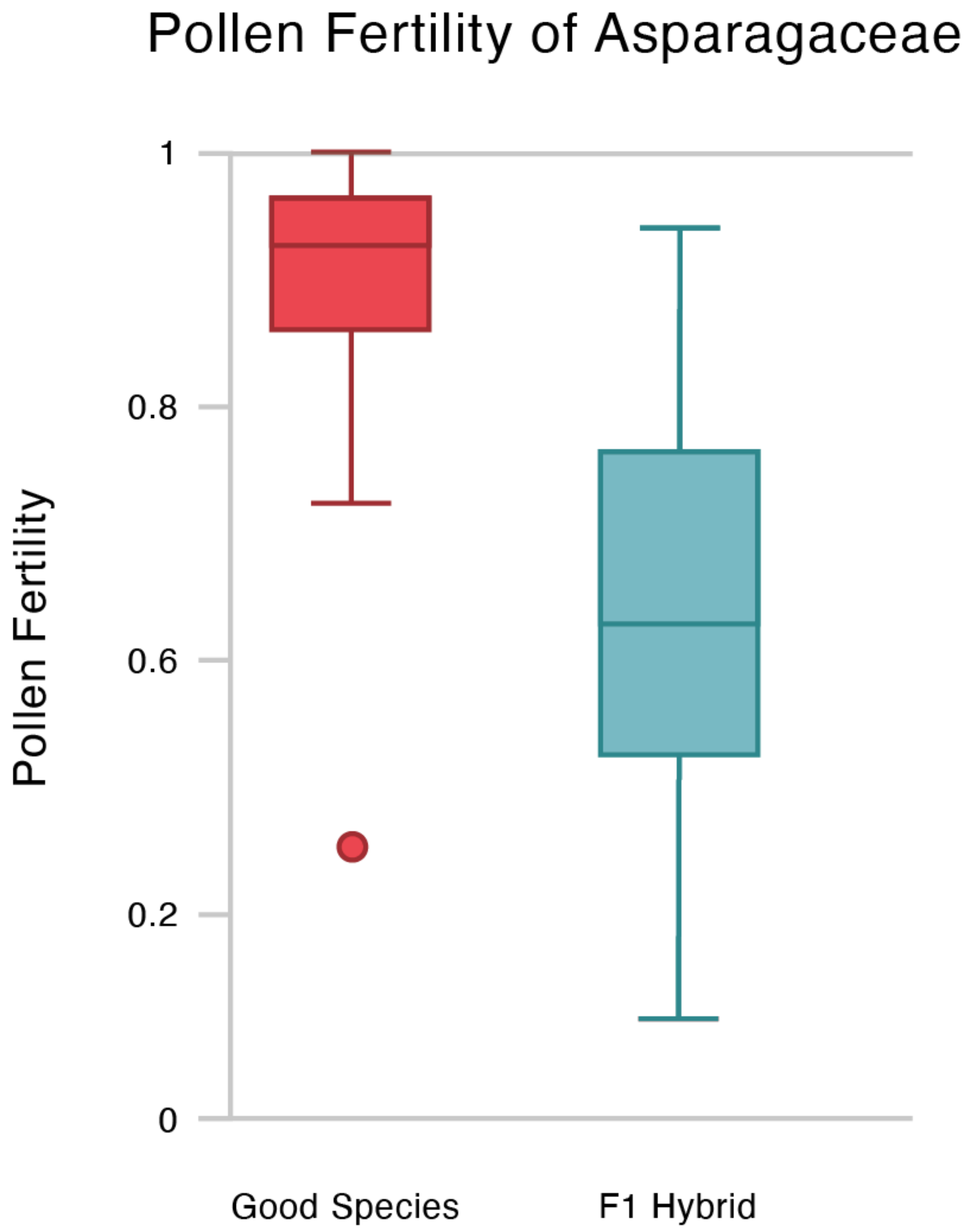

Supplementary Figure 29: Asparagaceae F1 hybrids (n=31) vs good species (n=30). Potential outliers labeled in red.

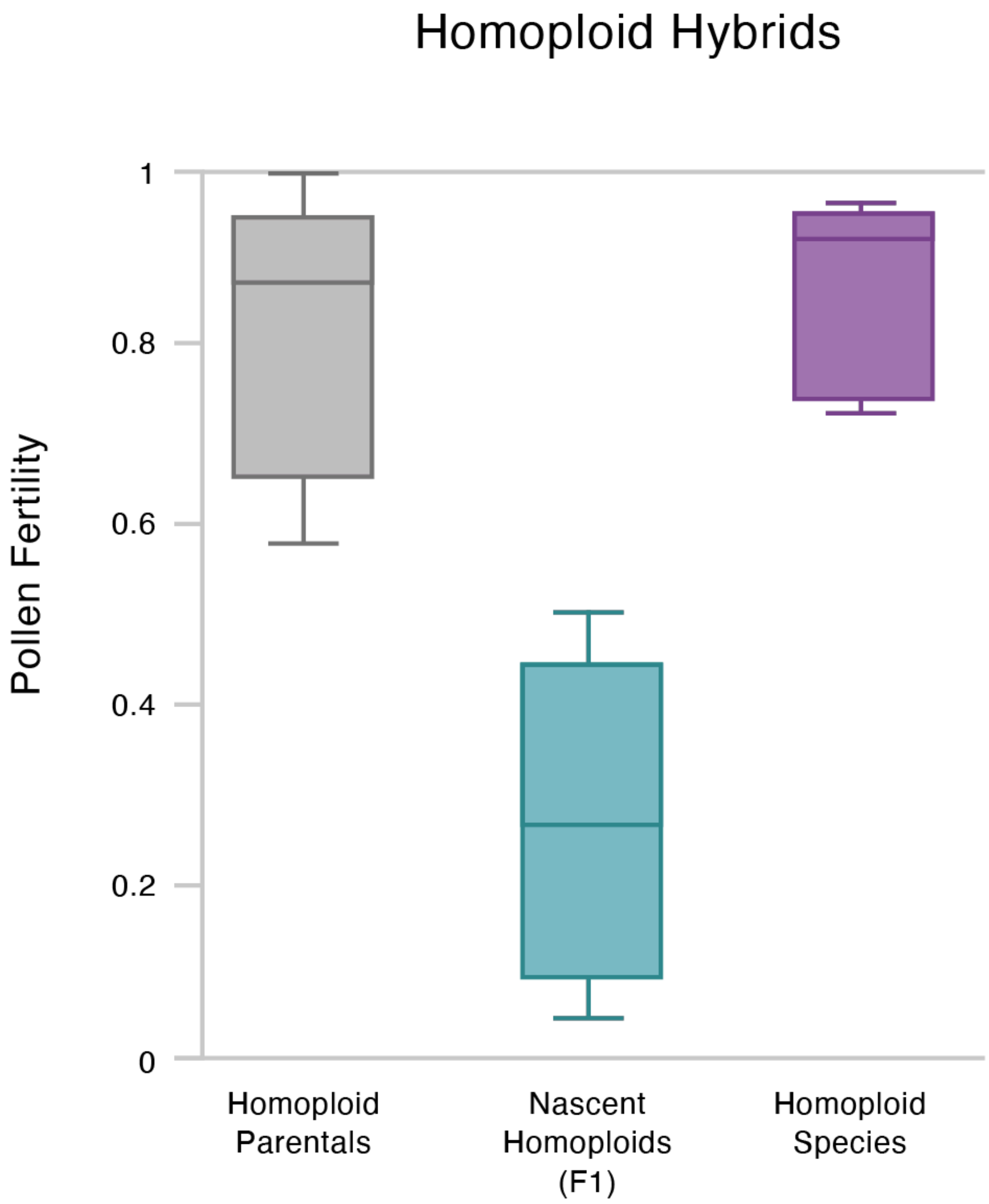

Supplementary Figure 30: Box plot of Homoploid hybrid parental species, nascent homoploid hybrids (F1s) and Homoploid Hybrid species. Note the the Homoploid parentals and Homoploid hybrid species have noticeably higher fertility than the F1s.

Pollen Fertility Comparison in F2s

Genus	Number of F1s	Good Species/F2s	Mean Hybrid Fertility	Mean Species/F2 Fertility	p-value
Dactylis	18	8	0.49	0.9	1.229e-9
F1-F2	18	13	0.49	0.34	1.422e-4 *
Dactylis-F2	13	8	0.34	0.9	5.091e-11*
Senecio	2	15	0.78	0.92	0.6063
F1-F2	2	2	0.78	0.9	0.6452
Senecio-F2	2	15	0.9	0.92	0.7826

Supplementary Table 1: Results from *Dactylis* and *Senecio* comparisons when F2 is included. The line with the genus name is the same as shown in main Table 1. The following two lines in each case document the comparisons between F1-F2 and true species and the F2.

Taxonomic Group	df	t-value	Lower 95% CI	Upper 95% CI	SD Hybrid	SD Species
Gillia	22	-9.3943*	-0.7293653	-0.4654595	0.21938	0.219578
Fuschia	17	-6.2596*	-0.7157826	-0.3554295	0.187383	0.193963

Elymus	8	-2.4239*	-1.04457153	-0.02313958	0.372499	0.40677
Helianthus	20	-10.394*	-0.7497112	-0.4990524	0.265314	0.031314
Passiflora	1	-2.1309	-1.950652	1.303489	0.210718	0.097106
Medicago	8	-2.1748	-0.190957066	0.004775248	0.047466	0.10906
Tolpis	14	-5.5234*	-0.7642881	-0.3357519	0.277079	0.13383
Cucumis	15	-6.1031	-0.6034477	-0.290825	0.19083	0.111096
Dactylis	19	-11.055*	-0.4911550	-0.3346367	0.108177	0.07719
Senecio	1	-0.70668	-2.422465	2.154585	0.266579	0.076583
Solanum	20	-6.8224*	-0.7247357	-0.3849437	0.302795	0.122566
Liliaceae	10	-4.4766*	-0.8479494	-0.2850642	0.325049	0.248526
Polemoniaceae	42	-11.984*	-0.7084355	-0.504275	0.21938	0.195098
Laminaceae	11	-5.1898*	-0.8313994	-0.3348386	0.366774	0.17892
Fabaceae	32	-8.0969*	-0.5620846	-0.3361469	0.280941	0.179503
Onagraceae	17	-5.342*	-0.7392258	-0.3210274	0.279167	0.196684
Asteraceae	277	-14.497*	-0.4990292	-0.3797046	0.35759	0.247895
Poaceae	107	-18.355	-0.6384279	-0.5139982	0.269797	0.157336
Saxifragaceae	6	-2.6841*	-0.58597939	-0.02732561	0.228658	0.144106

Brassicaceae	35	-6.6619*	-0.5965275	-0.3178049	0.368068	0.113512
Caryophyllaceae	7	-1.8729	-0.49636425	-0.05508092	0.328872	0.09276
Iridaceae	3	-12.005*	-1.027501	-0.608919	0.071418	0.204704
Malvaceae	7	-2.8783*	-0.53981872	0.05235271	0.262426	0.136337
Hydrangeaceae	7	-5.0451*	-0.6120838	-0.2193662	0.160643	0.161427
Curcurbitaceae	30	-6.2462*	-0.6334250	-0.3213179	0.304696	-0.140992
Ranunculaceae	68	-5.7181*	-0.2695861	-0.1301094	0.187779	0.13719
Rosaceae	2	-1.917	-0.803995	-0.2795257	0.231554	0.20502
Rubiaceae	2	-13.636*	-0.9505807	-0.460184	0.062933	0.153775
Asparagaceae	50	-7.2132*	-0.4503515	-0.2541894	0.230323	0.142174

Supplementary Table 2: Statistical data from all taxonomic groups species to F1 comparison. * indicate significant t-values at $\alpha < 0.05$.

**Genus**	**df**	**t-value**	**Lower 95% CI**	**Upper 95% CI**	**SD Hybrid**	**SD Species/ F2**
Dactylis	19	-11.055*	-0.4911550	-0.3346367	0.108177	0.07719

F1-F2	29	4.3792	0.07925107	0.21816688	0.108177	0.080865
Dactylis-F2	16	-15.899*	-0.6366777	-0.4865319	0.080865	0.07719
Senecio	1	-0.70668	-2.422465	2.154585	0.266579	0.076583
F1-F2	1	-0.6038	-2.038118	1.803718	0.266579	0.065478
Senecio-F2	1	-.03325	-0.3538356	0.3203556	0.065478	0.076583

Supplementary Table 3: Statistical data from *Dactylis* and *Senecio* F1 and F2 comparisons. The line with the genus name is the same as shown in Supplementary Table 2. The following two lines in each case document the comparisons between F1-F2 and true species and the F2 respectively. * indicates a significant t-value

Genus	df	t-value	Lower 95% CI	Upper 95% CI	SD Hybrid	SD Species
Homoploid Hybrid Species-Hybrid Progenitors	6	-4.2572*	-0.8587059	-0.2307941	0.210937	0.204937
Homoploid Hybrid Species-Ancestral Species	13	-0.041888	-0.1927924	0.1854724	0.204937	0.154426
Ancestral Species-Hybrid Progenitors	4	-4.7185*	-0.8609271	-0.2358929	0.210937	0.154426

Supplementary Table 4: Statistical data from the homoploid hybrid species compared to their parentals and to their progenitor hybrids. Note that the SD Species column contains the ancestral species when those are present in the comparison, the homoploid hybrid species when they are not.
